# Hydrothermal Assembly, Structural Multiplicity, and Catalytic Knoevenagel Condensation Reaction of a Series of Coordination Polymers Based on a Pyridine-Tricarboxylic Acid

**DOI:** 10.3390/molecules28227474

**Published:** 2023-11-08

**Authors:** Xiuqi Kang, Chao Ren, Zhenzhong Mei, Xiaoxiang Fan, Jijun Xue, Yongliang Shao, Jinzhong Gu

**Affiliations:** State Key Laboratory of Applied Organic Chemistry, College of Chemistry and Chemical Engineering, Lanzhou University, Lanzhou 730000, China; 220220923660@lzu.edu.cn (X.K.); rch31.nwnu@163.com (C.R.); meizhzh19@lzu.edu.cn (Z.M.); fanxx21@lzu.edu.cn (X.F.); xuejj@lzu.edu.cn (J.X.)

**Keywords:** coordination polymers, crystal structures, new topology, catalysis, Knoevenagel condensation

## Abstract

A pyridine-tricarboxylic acid, 5-(3′,5′-dicarboxylphenyl)nicotinic acid (H_3_dpna), was employed as a adjustable block to assemble a series of coordination polymers under hydrothermal conditions. The seven new coordination polymers were formulated as [Co(*μ*_3_*-*Hdpna)(*μ-*dpey)]*_n_*·*n*H_2_O (**1**), [Zn_4.5_(*μ*_6_*-*dpna)_3_(phen)_3_]*_n_* (**2**), [Co_1.5_(*μ*_6_*-*dpna)(2,2′-bipy)]*_n_* (**3**), [Zn_1.5_(*μ*_6_*-*dpna)(2,2′-bipy)]*_n_* (**4**), [Co_3_(*μ*_3_*-*dpna)_2_(4,4′-bipy)_2_(H_2_O)_8_]*_n_*·2*n*H_2_O (**5**),[Co(bpb)_2_(H_2_O)_4_]*_n_*[Co_2_(*μ*_3_*-*dpna)_2_(H_2_O)_4_]*_n_*·3*n*H_2_O (**6**), and [Mn_1.5_(*μ*_6_*-*dpna)(*μ-*dpea)]*_n_* (**7**), wherein 1,2-di(4-pyridyl)ethylene (dpey), 1,10-phenanthroline (phen), 2,2′-bipyridine(2,2′-bipy),4,4′-bipyridine(4,4′-bipy),1,4-bis(pyrid-4-yl)benzene (bpb), and 1,2-di(4-pyridyl)ethane (dpea) were employed as auxiliary ligands. The structural variation of polymers **1**–**7** spans the range from a 2D sheet (**1**–**4**, **6**, and **7**) to a 3D metal–organic framework (MOF, **5**). Polymers **1**–**7** were investigated as heterogeneous catalysts in the Knoevenagel condensation reaction, leading to high condensation product yields (up to 100%) under optimized conditions. Various reaction conditions, substrate scope, and catalyst recycling were also researched. This work broadens the application of H_3_dpna as a versatile tricarboxylate block for the fabrication of functional coordination polymers.

## 1. Introduction

Nowadays, a growing number of transition metal coordination polymers (CPs) serve as crystalline materials because their various topologies and structures [1,2,3]. These functional compounds have prospective applications in gas separation and adsorption [4,5,6], the development of sensors [7,8] and heterogeneous catalysts [9,10,11], and many other fields [12,13,14]. Solvothermal and hydrothermal synthetic methods are widely used in the assembly of coordination polymers [15,16,17,18]. In the process of self-assembly, many reaction parameters (pH, temperature, concentration, solvent, etc.) as well as the intrinsic characteristics of the organic ligands and metal centers [19,20,21,22,23,24,25,26] affect the structures of the coordination polymers.

**Scheme 1:**
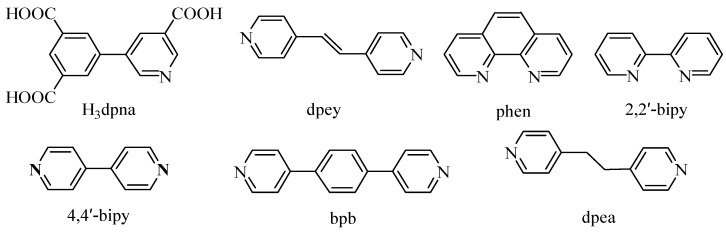
Structures of H_3_dpna and auxiliary ligands.

As the main kind of organic ligands for synthesizing coordination polymers, aromatic multi-carboxylic acids are especially attractive given their adjustable deprotonation and charges, tunable coordination modes, and good thermal stability [27,28,29,30]. Since 2019, our group has been working on the synthesis of coordination polymers with various carboxylic acids and exploring their catalytic performance in organic reactions [31,32,33]. Recently, our group used a pyridine-dicarboxylic acid, 4,4′-(pyridine-3,5-diyl)dibenzoic acid (H_2_pdba), to synthesize a series of transition metal coordination polymers. The catalytic performance of these polymers was evaluated [34]. As a continuation of that research, 5-(3′,5′-dicarboxylphenyl)nicotinic acid (H_3_dpna, Scheme 1) has been selected in the present study as a little studied pyridine-tricarboxylate building block.

As a linker, H_3_dpna has a lot of interesting characteristics that favor its exploration. (a) There is some rotation between the phenyl and pyridine rings, leading to improved adaptability to coordination preferences of metal(II) centers. (b) H_3_dpna bears some sites for coordination (six carboxyl O sites and one pyridine N site). (c) The presence of a free N_pyridine_ atom can provide a base site for catalysis, including Knoevenagel condensation [33,34]. (d) In 2017–2021, several Mn(II)-, Ni(II)-, Co(II)-, Zn(II)-, and Cd(II)-dpna coordination polymers were synthesized [35,36,37,38]. Although their magnetism, luminescence, selective adsorption, and proton conductivities were studied, the catalytic properties of only two H_3_dpna-based frameworks were investigated. Thus, the present work can aid in developing this field.

Hence, we report in this work the synthesis and analysis of seven new coordination polymers obtained from metal(II) salts, H_3_dpna, and different auxiliary ligands. The structures of these polymers vary from a 2D sheet (**1**–**4**, **6** and **7**) to a 3D metal–organic framework (MOF, **5**), namely [Co(*μ*_3_*-*Hdpna)(*μ-*dpey)]*_n_*·*n*H_2_O (**1**), [Zn_4.5_(*μ*_6_*-*dpna)_3_(phen)_3_]*_n_* (**2**), [Co_1.5_(*μ*_6_*-*dpna)(2,2′-bipy)]*_n_* (**3**), [Zn_1.5_(*μ*_6_*-*dpna)(2,2′-bipy)]*_n_* (**4**), [Co_3_(*μ*_3_*-*dpna)_2_(4,4′-bipy)_2_(H_2_O)_8_]*_n_*·2*n*H_2_O (**5**), [Co(bpb)_2_(H_2_O)_4_]*_n_*[Co_2_(*μ*_3_*-*dpna)_2_(H_2_O)_4_]*_n_*·3*n*H_2_O (**6**), and [Mn_1.5_(*μ*_6_*-*dpna)(*μ-*dpea)]*_n_* (**7**).

The catalytic properties of the obtained coordination polymers have also been evaluated in Knoevenagel reactions between benzaldehyde and malononitrile.

## 2. Results and Discussion

### 2.1. Hydrothermal Synthesis of Polymers ***1***–***7***

**Scheme 2:**
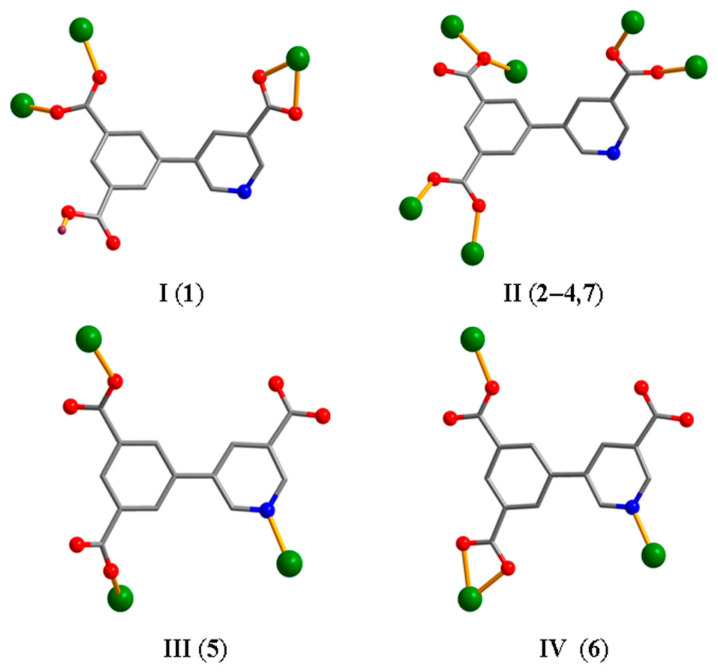
The coordination modes of Hdpna^2−^/dpna^3−^.

To evaluate 5-(3′,5′-dicarboxylphenyl)nicotinic acid (H_3_dpna) as a promising block for the fabrication of coordination polymers, some reactions were performed under hydrothermal conditions, using a mixture containing a metal(II) chloride, H_3_dpna (main ligand), sodium hydroxide, and an auxiliary ligand selected from dpey, phen, 2,2′-bipy, 4,4′-bipy, bpb, and dpea. These hydrothermal synthetic reactions were carried out at 160 °C for three days, and then continued through a slow cooling of the mixtures and the crystallization of coordination polymers, as described in Table 1. The polymers were collected in 42–53% yields and characterized using standard methods. The obtained PXRD patterns of **1**–**7** are consistent with the simulated ones (Figure S3), confirming the formation of the homogeneous phase of the obtained polymers. The structural differences in **1**–**7** might be attributed to the type of auxiliary ligand selected as well as the coordination fashion of the metal(II) centers. In polymers **1**–**7**, the blocks generated upon the deprotonation of H_3_dpna showed four different coordination fashions and are partially or totally deprotonated (Hdpna^2−^and dpna^3−^, Scheme 2).

### 2.2. Crystal Structure of ***1***

**Figure 1:**
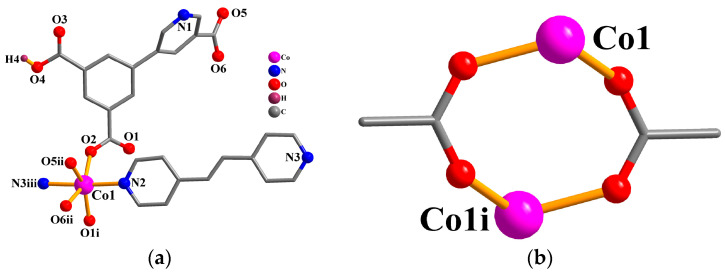

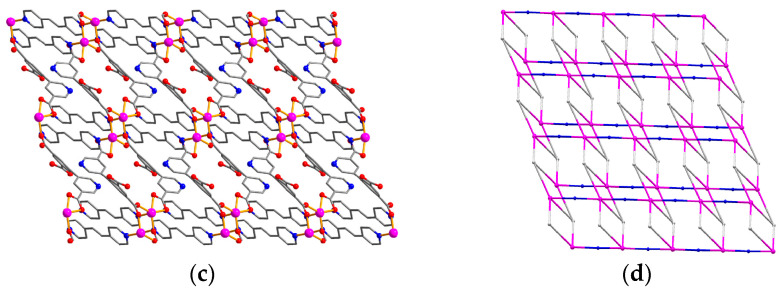
Structure of **1**. (**a**) Coordination environment at Co(II) atom; hydrogen atoms are ignored except for COOH group. (**b**) The Co_2_ subunit. (**c**) The 2D sheet (view down the *c* axis). (**d**) Topological representation of a 3-connected network with topology (view down the *c* axis); 5-connected Co1 nodes (pink), centroids of 3-connected *µ*_3_-Hdpna^2−^ nodes (gray), centroids of 2-connected *µ*-dpey linkers (blue).

As depicted in Figure 1a, there are one Co(II) center, one *μ*_3_*-*Hdpna^2−^ block, one *μ-*dpey auxiliary ligand, and one free water molecule in the asymmetric unit of polymer **1**. The six-coordinate Co1 center possesses a distorted octahedral {CoN_2_O_4_} environment populated by four carboxyl oxygen atoms from three *μ*_3_-Hdpna^2^ linkers and two N donors from two different *μ*-dpey moieties. The bond lengths of Co–O and Co–N are 2.017(2)–2.246(2) and 2.154(3)–2.156(2) Å, respectively; these are within the normal ranges observed in related Co(II) compounds [7,13]. In **1**, the Hdpna^2^ ligand takes the coordination model (Scheme 2) with two COO^−^ groups being bidentate or bridging bidentate, while the nitrogen site of the pyridine ring remains uncoordinated. Two carboxylate groups of two *μ*_3_*-*Hdpna^2−^ linkers connect the adjacent Co(II) centers to give a dimeric Co_2_ subunit with a Co∙∙∙Co distance of 4.021(3) Å (Figure 1b). These Co_2_ subunits are further connected by the *µ*_3_-Hdpna^2−^ linkers and *μ-*dpey ligands into a 2D network (Figure 1c). This 2D structure is composed of the 5-linked Co1 nodes, 3-linked *µ*_3_-Hdpna^2−^ nodes, and 2-connected *µ*-dpey linkers (Figure 1d). This is a topology and a point symbol of (4^2^.6^7^.8)(4^2^.6).

### 2.3. Crystal Structure of ***2***

**Figure 2:**
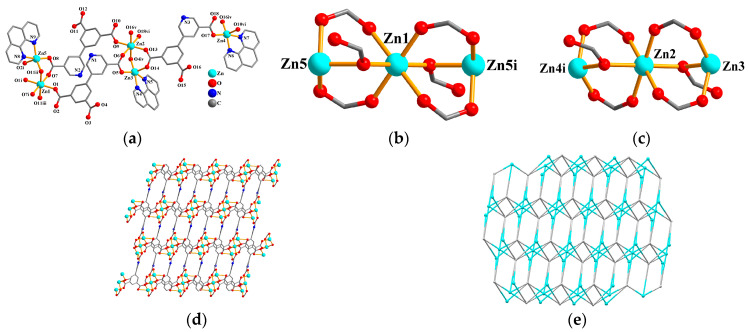
Structure of **2**. (**a**) Coordination environment at Zn(II) centers; hydrogen atoms are ignored. (**b**,**c**) Zn_3_ subunits; the phen moieties are ignored. (**d**) The 2D sheet (view down the *b* axis). (**e**) Topological representation of a 2-nodal network with topology (view down the *b* axis); Zn series nodes (cyan), centroids of 6-linked *µ*_6_-dpna^3−^ nodes (gray).

The asymmetric unit of **2** consists of five distinct Zn(II) centers (Zn1 with 1/2 occupancy; Zn2–Zn5 with full occupancy), three *μ*_6_*-*dpna^3−^ blocks, and three phen supporting ligands. The Zn1 and Zn2 atoms are six-coordinate and feature distorted octahedral {ZnO_6_} environments (Figure 2a). The Zn3, Zn4, and Zn5 centers are five-coordinate and display distorted trigonal bipyramidal {ZnN_2_O_3_} geometries. The Zn–O [2.003(2)–2.187(2) Å] and Zn–N [2.097(2)–2.214(2)Å] distances are comparable to those in Zn(II) derivatives [5,6,12]. In **2**, the dpna^3−^ blocks behave as *μ*_6_-spacers (mode II, Scheme 2), in which the COO^−^ groups exhibit bridging bidentate modes, while the N donor of the pyridine ring remains uncoordinated. The three adjacent Zn(II) atoms (Zn1, Zn5, and Zn5i) are clustered by means of four carboxylate groups and two carboxyl oxygen atoms of six different *μ*_6_*-*dpna^3−^ blocks, generating a trizinc subunit (Figure 2b). Meanwhile, another Zn_3_ (Zn2, Zn3, and Zn4i) subunit is formed (Figure 2c). These Zn_3_ subunits are further assembled by the remaining carboxylate groups of the *µ*_6_-dpna^3−^ blocks to give a 2D network (Figure 2d). The 2D network in this structure is built from the 3- and 6-linked Zn1/Zn2 and 6-linked *µ*_6_-dpna^3−^ nodes (Figure 2e). It can be defined as a trinodal net with a topology and point symbol of (4^12^.6^3^)(4^3^)_2_(4^6^.6^9^)_2_.

### 2.4. Crystal Structures of ***3*** and ***4***

**Figure 3:**
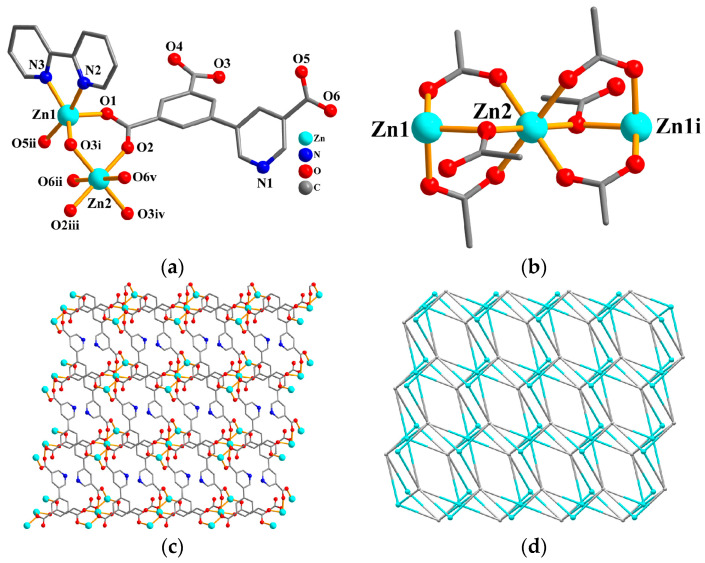
Structure of **4**. (**a**) Coordination environment of Zn(II) atoms; hydrogen atoms are ignored. (**b**) The Zn_3_ subunit. (**c**) Two-dimensional metal–organic network (view down the *c* axis). (**d**) Topological representation of a 3,6-connected network with topology (view along the *c* axis); 3- and 6-linked Zn1/Zn2 nodes (cyan), centroids of 6-connected *µ*_6_-dpna^3−^ nodes (gray).

Since coordination polymers **3** and **4** are isostructural, only polymer **4** is described herein (Figure 3). The asymmetric unit of Zn(II) polymer **4** bears two Zn(II) atoms (Zn1 with full occupancy, Zn2 with 1/2 occupancy), one *µ*_6_-dpna^3−^block, and one 2,2′-bipy auxiliary ligand (Figure 3a). The five-coordinate Zn1 atom possesses a distorted trigonal bipyramidal {ZnN_2_O_3_} environment, which is formed by three carboxyl oxygen atoms from three distinct *µ*_6_-dpna^3−^ blocks and a pair of N_2,2′-bipy_ donors. The Zn2 center is six-coordinate and assumes as a distorted octahedral {ZnO_6_} environment, based on six carboxyl oxygen atoms coming from six different *µ*_6_-dpna^3−^ blocks. The Zn–O [2.018(2)–2.134(2) Å] and Zn–N [2.100(3)–2.164(3) Å] bonds are within standard values [13,17,26]. The dpna^3−^ block acts as a *µ*_6_-linker (mode II, Scheme 2). The Zn_3_ subunit is found (Figure 3b), which is similar to that in polymer **2**. These Zn_3_ subunits are further assembled by the other carboxylate groups of the µ_6_-dpna^3−^ blocks to produce a 2D network (Figure 3c). This 2D structure is composed of the 3- and 6-linked Zn1/Zn2 nodes, and 6-linked *µ*_6_-dpna^3−^ nodes (Figure 3d). It is a topology and a point symbol of (4^12^.6^3^)(4^3^)_2_(4^6^.6^9^)_2_.

### 2.5. Crystal Structure of ***5***

**Figure 4:**
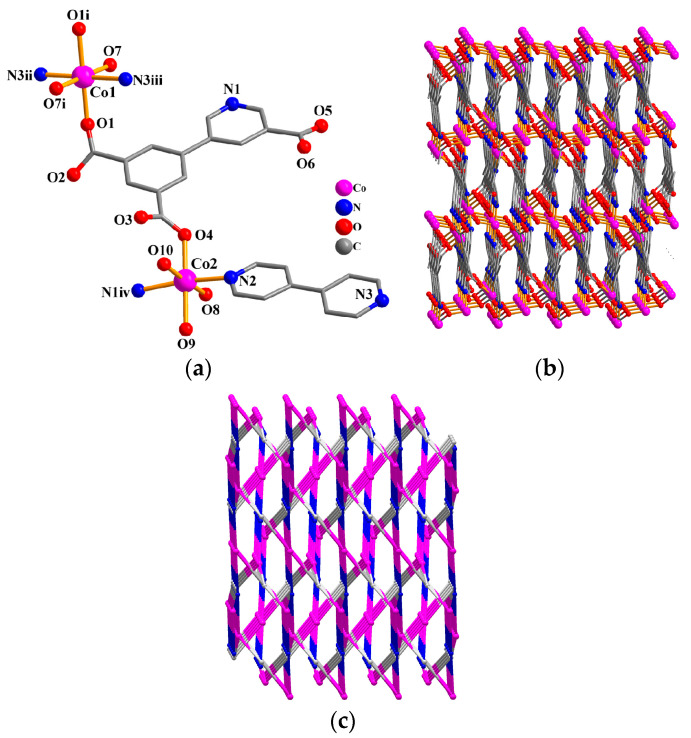
Structure of **5**. (**a**) Coordination environments at Co(II) atoms; hydrogen atoms are ignored. (**b**) Three-dimensional metal–organic framework (view down the *c* axis). (**c**) Topological representation of a 2,3,4-connected framework with topology (view down the *c* axis); 3- and 4-linked Co1/Co2 nodes (pink), centroids of 3-connected *µ*_3_-dpna^3−^ nodes (gray), centroids of 2-connected *µ*-4,4′-bipy linkers (blue).

Compound **5** is a 3D coordination polymer (Figure 4) and its asymmetric unit reveals two Co(II) centers (Co1 with 1/2 occupancy, Co2 with full occupancy), one *µ*_3_-dpna^3−^ block, one *µ*-4,4′-bipy moiety, four H_2_O ligands, and one free water molecule (Figure 4a). As shown in Figure 4a, the Co1 center is six-coordinate and forms a distorted octahedral {CoN_2_O_4_} geometry. It is completed by two carboxyl oxygen atoms from two *μ*_3_*-*dpna^3−^, two O donors from two H_2_O ligands, and two N_4,4′-bipy_ from two *µ*-4,4′-bipy moieties. The six-coordinate Co2 atom also displays a distorted octahedral {CoN_2_O_4_} geometry filled by one carboxyl oxygen atom from one μ_3_-dpna^3−^, two N donors from two distinct *µ*-4,4′-bipy moieties, and three O atoms of three H_2_O ligands. The Co–O [2.078(2)–2.131(2) Å] and Co–N [2.157(2)–2.160(2) Å] bonds are within standard values [17,21,22]. The dpna^3−^ moiety adopts a *μ*_3_*-*coordination fashion (mode III, Scheme 2), in which COO^−^ groups exhibit monodentate or uncoordinated fashions; the N donor of the pyridine ring is coordinated to the Co(II) center. The *μ*_3_-dpna^3−^ blocks and *µ*-4,4′-bipy moieties form multiple connections to the Co centers to form a 3D framework (Figure 4b). The 3D network is assembled from the 3- and 4-linked Co1/Co2 centers, the 3-connected *µ*_3_-dpna^3−^ nodes, and 2-connected *µ*-4,4′-bipy linkers (Figure 4c). The resulting network possesses a topology with a net point symbol of (6.10^2^)_4_(6^2^.12^4^).

### 2.6. Crystal Structure of ***6***

**Figure 5:**
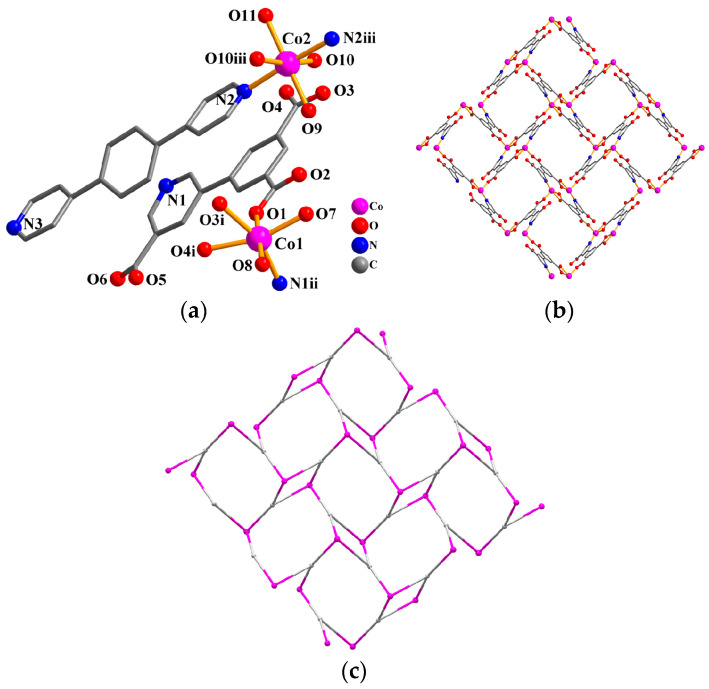
Structure of **6**. (**a**) Coordination environment at Co(II) atoms; hydrogen atoms are ignored. (**b**) Two-dimensional cation metal–organic network (view down the *a* axis). (**c**) Topological representation of a 3-connected network with a **fes** topology (view down the *a* axis); 3-connected Co1 nodes (pink), centroids of 3-connected *µ*_3_-dpna^3−^ nodes (gray).

The crystal structure of compound **6** is composed of one cation [Co(bpb)_2_(H_2_O)_4_]^2+^, one anion 2D sheet [Co_2_(*μ*_3_*-*dpna)_2_(H_2_O)_4_]^2−^*_n_*, and three lattice water molecules (Figure 5a). In the cation, the Co2 center adopts a distorted octahedral {CoN_2_O_4_} environment filled by four oxygen atoms from the four H_2_O ligands and a pair of N donors of two individual bpb auxiliary ligands. In the cation 2D sheet, the Co1 center is also six-coordinate and displays a distorted octahedral {CoNO_5_} environment, which is constituted by three carboxyl oxygen and nitrogen atoms from four different *μ*_3_*-*dpna^3−^ blocks and two oxygen donors from two H_2_O ligands. The Co–O [2.048(3)–2194(2) Å] and Co–N [2.102(2)–2.141(2) Å] distances are comparable to those in related Co(II) derivatives [23,24,25]. The dpna^3−^ block acts as a *µ*_3_-linker (mode IV, Scheme 2) with three COO^−^ groups being monodentate, bidentate, or uncoordinated; the N donor of the pyridine ring is coordinated to the Co(II) center. The bpb auxiliary ligand shows a terminal coordination fashion. The *µ*_3_-dpna^3−^ blocks link to the Co1 centers to form a cation 2D metal–organic network (Figure 5b). This 2D structure is composed of the 3-linked Co1 nodes and 3-linked *µ*_3_-dpna^3−^ nodes (Figure 5c). It is a **fes** topology with a point symbol of (4.8^2^).

### 2.7. Crystal Structure of ***7***

**Figure 6:**
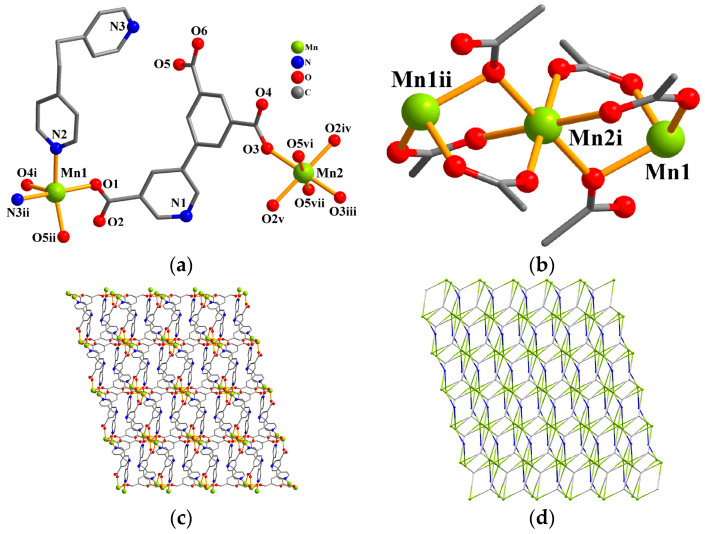
Structure of **7**. (**a**) Coordination environment at Mn(II) atoms; hydrogen atoms are ignored. (**b**) Mn_3_ unit. (**c**) Two-dimensional metal–organic network (view down the *c* axis). (**d**) Topological representation of a 2,5,6-connected network with topology (view down the *c* axis); Mn series nodes (light green), centroids of 6-connected *µ*_6_-dpna^3−^ nodes (gray), centroids of 2-connected *μ-*dpea linkers (blue).

This compound has a 2D metal–organic network structure. Its asymmetric unit contains two distinct Mn(II) centers (Mn1 with full occupancy; Mn2 with 1/2 occupancy and located on a 2-fold rotation axis), a *µ*_6_-dpna^3−^ block, and a bridging dpea ligand. The Mn1 atom is five-coordinated and forms a distorted trigonal bipyramidal {MnN_2_O_3_} geometry, which is completed by three carboxyl oxygen donors coming from three distinct µ_6_-dpna^3−^ moieties and two N donors from two dpea ligand (Figure 6a). The Mn2 center is six-coordinate and positioned on a 2-fold rotation axis; Mn2 is surrounded by six O donors from six distinct *µ*_6_-dpna^3−^ blocks, thus forming a distorted octahedral {MnO_6_} environment. The Mn–O [2.112(2)–2.178(2) Å] and Mn–N [2.207(2)–2.313(2)Å] lengths are comparable to those in related Mn(II) derivatives [23,26,32]. In **7**, the dpna^3−^ block behaves as a *μ*_6_-spacer (Scheme 2, mode II), in which the COO^−^ groups exhibit *μ*-bridging bidentate modes. Two Mn1 and one Mn2 centers are bridged by four carboxylate groups and two carboxyl O atoms from six different *µ*_6_-dpna^3−^ blocks, thus forming a centrosymmetric trimanganese(II) cluster (Figure 6b). These Mn_3_ clusters are additionally interlinked by the *µ*_6_-dpna^3−^ blocks to give an intricate 2D metal–organic layer (Figure 6c). This 2D structure is composed of the 5- and 6-linked Mn1/Mn2 nodes, 6-linked *µ*_6_-dpna^3−^ nodes, and 2-connected *μ-*dpea linkers (Figure 6d). It is a topology and a point symbol of (3.4^6^.5^6^.6^2^)_2_(3^2^.4^3^.5^4^.6)_2_(4^12^.6^3^).

### 2.8. TGA and PXRD Data

In an atmosphere of nitrogen, the thermal stability of polymers **1**–**7** were investigated by TGA (Figure S2) in the range of 21–800 °C. In compound **1**, one lattice water molecule was lost at 157–192 °C (exptl 3.2%, calcd 3.3%), and the dehydrated sample was stable up to 324 °C. Polymers **2**–**4** and **7** do not contain free solvent or coordinated H_2_O, and were stable until 382, 347, 383, and 373 °C, respectively. In **5**, two lattice water molecule and eight water ligands were lost at 140–240 °C (exptl, 14.2%; calcd, 14.5%), and the dehydrated framework was stable up to 265 °C. For polymer **6**, a loss of mass at 51–152 °C was attributed to the loss of three lattice and eight coordinated water molecules (exptl, 14.3%; calcd, 14.1%), while the sample maintained stability until 335 °C.

For all the obtained products **1**–**7**, power X-ray diffractograms were collected at 25 °C (Figure S3). The comparison between the experimental patterns and the simulated ones (coming from CIF data) showed that all samples are pure.

### 2.9. Catalytic Knoevenagel Condensation

**Scheme 3:**
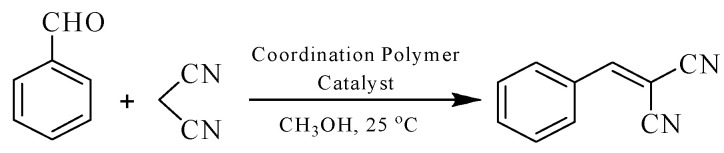
Knoevenagel condensation of benzaldehyde with malononitrile.

Given the ability of some coordination polymers to catalyze the Knoevenagel condensation reaction of aldehydes and active methylene compounds [39,40,41], the reaction was performed to evaluate the catalytic activities of polymers **1**–**7**. Firstly, benzaldehyde and malononitrile were employed as reagents and catalytic experiments were carried out in CH_3_OH at 25 °C, leading to the generation of 2-benzylidenemalononitrile (Scheme 3, Table 2). The influence of important parameters on the reaction yield was also studied, including the time, solvent, temperature, and amount of catalyst.

Preliminary screening of all the obtained polymers revealed that compounds **2**–**4** and **7** were the most promising. Because polymer **4** had a higher yield when it was synthesized, this polymer was used to further optimize the reaction parameters. The reaction time had a great influence on the yield; the product yield increased from 45 to 100% (Table 2, entries 1–6; Figure S4) when the reaction was extended from 10 min to 60 min. The influence of catalyst amount was also investigated, resulting in an increase in product yield from 94 to 100% when the catalyst amount was increased from 1 to 2 mol% (Table 2, entries 6 and 7). Although the highest reaction yield was achieved in CH_3_OH, water, ethanol, acetonitrile, and chloroform were also used as alternative solvents, leading to lower product yields (65–98%). In the absence of the above catalysts, the catalytic reaction was not efficient (Table 2, entries 18–20). Although no correlation between catalytic performance and the structure of coordination polymer catalyst could be concluded, the superior performance of polymers **2**–**4** and **7** can be attributed to the existence of the non-saturated coordination site in the metal centers [42,43].

Next, some benzaldehyde substrates bearing functional groups were reacted under the best conditions (2.0 mol% **4**, CH_3_OH, 60 min). The respective products of the reaction were obtained in yields ranging from 33 to 100% (Table S3). Benzaldehyde and tis derivatives bearing an electron-attracting group (e.g., –NO_2_ or –Cl) showed a higher reactivity (Table S3, entries 1–5). This could be attributed to an increased electrophilic characteristic of the above benzaldehydes. Benzaldehyde substrates bearing electron-donor groups (e.g., –CH_3_ or –OCH_3_) had lower yields of products (Table S3, entries 6 and 7).

Catalyst recycling tests (Figure S5) confirm that polymer **4** maintains its catalytic activity during five cycles, which was borne out by the similarity of product yields. Moreover, the PXRD data also support the maintenance of the structure of catalyst **4** (Figure S6).

## 3. Experiments

### 3.1. Materials and Measurements

Except where otherwise noted, all chemicals were commercially available and were used as received. H_3_dpna was acquired from Yanshen Tec. Co., Ltd. (Changchun, China). A Bruker EQUINOX 55 spectrometer (Bruker Corporation, Billerica, MA, USA) was used to record the FTIR spectra (KBr discs). The C, H, and N elemental analyses (EA) for **1**–**7** were conducted using an Elementar Vario EL elemental analyzer (Elementar, Langenselbold, Germany). An LINSEIS STA PT1600 thermal analyzer (Linseis Messgeräte GmbH, Selb, Germany) was used for the thermogravimetric analyses (TGAs) under a N_2_ flow with a heating rate of 10 °C/min. A Rigaku-Dmax 2400 diffractometer (Cu-Kα radiation; *λ*= 1.54060 Å, Rigaku Corporation, Tokyo, Japan) was used to collect the powder X-ray diffraction (PXRD) patterns of the obtained compounds. CDCl_3_ was used as the solvent for ^1^H NMR measurements on a JNM ECS 400 M spectrometer (Bruker BioSpin AG, Fällanden, Switzerland).

### 3.2. Synthesis of [Co(μ_3_-Hdpna)(μ-dpey)]_n_·nH_2_O (***1***)

H_3_dpna (57.4 mg, 0.2 mmol), CoCl_2_·6H_2_O (47.6 mg, 0.2 mmol), dpey (36.4 mg, 0.2 mmol), NaOH (16.0 mg, 0.4 mmol), and H_2_O (10 mL) were sealed into a 20 mL Teflon cup. After the resulting mixture was kept at 160 °C for three days, it was cooled to room temperature, and pink block-shaped crystals were picked out and washed with water. (yield: 45% based on H_3_dpna). Calculated for C_26_H_19_CoN_3_O_7_: C 57.36, N 7.72, H 3.52%. Found: C 57.66, N 7.63, H 3.50%. FTIR (KBr, cm^−1^): 3463 w, 3043 w, 1708 m, 1608 s, 1589 s, 1437 s, 1405 s, 1325 w, 1288 w, 1265 w, 1205 w, 1176 w, 1136 w, 1073 w, 1017 w, 989 w, 901 w, 838 m, 793 w, 765 w, 701w, 625 w, 562 w.

### 3.3. Synthesis of [Zn_4.5_(μ_6_-dpna)_3_(phen)_3_]_n_ (***2***)

H_3_dpna (57.4 mg, 0.2 mmol), ZnCl_2_ (40.9 mg, 0.3 mmol), phen (59.4 mg, 0.3 mmol), NaOH (24.0 mg, 0.6 mmol), and H_2_O (10 mL) were sealed into a 20 mL Teflon cup. After the resulting mixture was kept at 160 °C for three days, it was cooled to room temperature, and colorless block-shaped crystals were picked out and washed with water (yield: 47% based on H_3_dpna). Calculated for C_78_H_42_Zn_4.5_N_9_O_18_: C 55.51, N 7.47, H 2.51%. Found: C 55.23, N 7.44, H 2.53%. FTIR (KBr, cm^−1^): 1628 m, 1608 m, 1577 w, 1516 w, 1425 s, 1345 s, 1305 w, 1277 w, 1176 w, 1145 w, 1102 w, 1026 w, 933 w, 905 w, 845 w, 777 m, 726 m, 686 w, 641 w, 565 w, 538 w.

### 3.4. Synthesis of [Co_1.5_(μ_6_-dpna)(2,2′-bipy)]_n_ (***3***)

H_3_dpna (57.4 mg, 0.2 mmol), CoCl_2_·6H_2_O (71.4 mg, 0.3 mmol), 2,2′-bipy (46.8 mg, 0.3 mmol), NaOH (24.0 mg, 0.6 mmol), and H_2_O (10 mL) were sealed into a 20 mL Teflon cup. After the resulting mixture was kept at 160 °C for three days, it was cooled to room temperature, and pink block-shaped crystals were picked out and washed with water (yield: 44% based on H_3_dpna). Calculated for C_24_H_14_Co_1.5_N_3_O_6_: C 54.51, N 7.95, H 2.67%. Found: C 54.73, N 7.74, H 2.68%. FTIR (KBr, cm^−1^): 1625 s, 1601 s, 1473 w, 1425 s, 1361 s, 1305 w, 1276 w, 1185 w, 1154 w, 1101 w, 1054 w, 1026 w, 973 w, 933 w, 897 w, 833 w, 762 m, 721 m, 686 w, 650 w, 629 w, 565 w, 534 w.

### 3.5. Synthesis of [Zn_1.5_(μ_6_-dpna)(2,2′-bipy)]_n_ (***4***)

Compound **4** was synthesized following a procedure similar to **3** except using ZnCl_2_ (40.9 mg, 0.3 mmol) instead of CoCl_2_·6H_2_O. Some colorless block-shaped crystals of compound **4** were collected (yield: 53% based on H_3_dpna). Calculated for C_48_H_28_Zn_3_N_6_O_12_: C 53.53, N 7.80, H 2.62%. Found: C 53.27, N 7.77, H 2.64%. FTIR (KBr, cm^−1^): 1632 s, 1608 s, 1476 w, 1425 s, 1376 w, 1340 w, 1276 w, 1181 w, 1149 w, 1102 w, 1053 w, 1021 w, 941 w, 902 w, 838 w, 790 m, 769 w, 729 m, 686 w, 650 w, 626 w, 565 w, 530 w.

### 3.6. Synthesis of [Co_3_(μ_3_-dpna)_2_(4,4′-bipy)_2_(H_2_O)_8_]_n_·2nH_2_O (***5***)

H_3_dpna (57.4 mg, 0.2 mmol), CoCl_2_·6H_2_O (71.4 mg, 0.3 mmol), 4,4′-bipy (46.8 mg, 0.3 mmol), NaOH (24.0 mg, 0.6 mmol), and H_2_O (10 mL) were sealed into a 20 mL Teflon cup. After the resulting mixture was kept at 160 °C for three days, it was cooled to room temperature, and pink needle-shaped crystals were picked out and washed with water (yield: 42% based on H_3_dpna). Calculated for C_24_H_24_Co_1.5_N_3_O_11_: C 46.58, N 6.79, H 3.91%. Found: C 46.83, N 6.75, H 3.93%. FTIR (KBr, cm^−1^): 3283 m, 1613 s, 1565 s, 1401 s, 1365 s, 1281 w, 1229 w, 1185 w, 1137 w, 1069 w, 1029 w, 1009 w, 897 w, 821 m, 789 w, 701 m, 634 w, 526 w.

### 3.7. Synthesis of [Co(bpb)_2_(H_2_O)_4_]_n_[Co_2_(μ_3_-dpna)_2_(H_2_O)_4_]_n_·3nH_2_O (***6***)

H_3_dpna (57.4 mg, 0.2 mmol), CoCl_2_·6H_2_O (71.4 mg, 0.3 mmol), bpb (69.7 mg, 0.3 mmol), NaOH (24.0 mg, 0.6 mmol), and H_2_O (10 mL) were sealed into a 20 mL Teflon cup. After the resulting mixture was kept at 160 °C for three days, it was cooled to room temperature, and red block-shaped crystals were picked out and washed with water (yield: 45% based on H_3_dpna). Calculated for C_60_H_58_Co_3_N_6_O_23_: C 51.18, N 5.97, H 4.15%. Found: C 51.35, N 6.01, H 4.13%. FTIR (KBr, cm^−1^): 3404 m, 3227 m, 3027 w, 1665 w, 1601 s, 1557 m, 1469 m, 1413 s, 1369 s, 1297 w, 1221 w, 1197 w, 1154 w, 1117 w, 1065 w, 1014 w, 909 w, 802 m, 781 m, 717 m, 658 w, 574 w.

### 3.8. Synthesis of [Mn_1.5_(μ_6_-dpna)(μ-dpea)]_n_ (***7***)

H_3_dpna (57.4mg, 0.2 mmol), MnCl_2_·4H_2_O (59.4 mg, 0.3 mmol), dpea (55.2 mg, 0.3 mmol), NaOH (24.0 mg, 0.6 mmol), and H_2_O (10 mL) were sealed into a 20 mL Teflon cup. After the resulting mixture was kept at 160 °C for three days, it was cooled to room temperature, and yellow block-shaped crystals were picked out and washed with water (yield: 47% based on H_3_dpna). Calculated for C_52_H_36_Mn_3_N_6_O_12_: C 56.69, N 7.63, H 3.29%. Found: C 56.94, N 7.47, H 3.31%. FTIR (KBr, cm^−1^): 1608 s, 1569 s, 1501 w, 1421 s, 1353 s, 1310 w, 1281 w, 1229 w, 1181 w, 1149 w, 1105 w, 1069 w, 1021 w, 902 w, 833 w, 786 m, 722 m, 681 w, 586 w, 553 w.

### 3.9. Single Crystal X-ray Diffraction and Topological Analysis

The crystal data of polymers **1**–**7** were collected by a Bruker APEX-II CCD diffractometer (graphite–monochromated Mo/Cu*K*_α_ radiation; *λ* = 0.71073/1.54184 Å). The structures were solved by direct methods and refined by full-matrix least-squares on *F*^2^ with SHELXS-97 and SHELXL-97 [44]. The C, O, and N atoms were refined anisotropically using full-matrix least-squares methods on *F^2^*. The H atoms were placed in calculated positions through riding models. In **2**, the phen ligands (N4, C43–C54, N5) were split over two sites and refined with 0.70 and 0.30 occupancies. The crystal parameters and structural refinements are summarized in Table 1. The selected bond parameters are collected in Tables S1 and S2 (Supplementary Materials). CCDC 2299559–2299565 contains the supplementary crystallographic data of **1**–**7**.

Topological analysis of the obtained coordination polymers was performed with ToposPro software (Version 5.5.2.1) by producing abbreviated (underlying) nets, therein bridging ligands were simplified to the centroids [45,46].

### 3.10. Catalytic Activity in Knoevenagel Condensation Reaction

At 25 °C, a suspension containing catalyst (typically 2 mol%), aromatic aldehyde (0.50 mmol, benzaldehyde as a model substrate), malononitrile (1.0 mmol), and solvent (1.0 mL, typically CH_3_OH) was stirred for the desired reaction time. Then, the catalyst was removed by centrifugation. The filtrate was evaporated using a rotary evaporator to obtain a crude solid product. This was dissolved in CDCl_3_ and analyzed by ^1^H NMR spectroscopy (JNM ECS 400 M spectrometer) for the quantification of the product (Figure S7). In order to run recycling tests, the catalyst was recollected by centrifugation, washed with methanol, dried at room temperature, and reused in subsequent steps as depicted above.

## 4. Conclusions

The present work revealed the application of H_3_dpna as a pyridine-tricarboxylate block for assembling novel coordination polymers. As a result, seven new coordination polymers were synthesized using the hydrothermal method. The structures of polymers **1**–**7** ranged from a 2D network (**1**–**4**, **6** and **7**) to metal–organic framework (**5**). The catalytic application of polymers **1**–**7** was evaluated in Knoevenagel reactions involving benzaldehydes and malononitrile. Polymers **2**–**4** and **7** revealed excellent catalytic performance in the Knoevenagel reaction of aldehydes with malononitrile.

## Figures and Tables

**Table 1:** Summary of crystal data for compounds **1**–**7**.

Compound	1	2	3	4
Chemical formula	C_26_H_19_CoN_3_O_7_	C_78_H_42_Zn_4.5_N_9_O_18_	C_24_H_14_Co_1.5_N_3_O_6_	C_48_H_28_Zn_3_N_6_O_12_
Molecular weight	544.37	1687.37	528.78	1076.87
Crystal system	Triclinic	Triclinic	Triclinic	Triclinic
Space group	*P* − 1	*P* − 1	*P* − 1	*P* − 1
*a*/Å	9.3127(5)	10.2995(2)	9.52279(17)	9.5341(4)
*b/*Å	12.2573(5)	14.4165(3)	10.17273(19)	10.2025(3)
*c*/Å	12.3293(6)	23.8214(5)	12.5987(2)	12.6077(5)
*α/*(°)	119.515(5)	88.9997(15)	76.6446(16)	76.362(3)
*β*/(°)	103.370(4)	81.6471(16)	71.2712(17)	70.953(4)
*γ/*(°)	97.284(4)	84.2605(16)	78.5857(16)	78.498(3)
*V*/Å^3^	1142.36(11)	3481.98(12)	1114.40(4)	1116.49(8)
*Z*	2	2	2	1
*F*(000)	558	1704	535	544
Crystal size/mm	0.09 × 0.03 × 0.03	0.06 × 0.04 × 0.03	0.09 × 0.05 × 0.04	0.10 × 0.07 × 0.05
*θ* range for data collection	1.991–25.50	1.871–75.71	3.766–67.982	2.073–25.999
Limiting indices	−11 ≤ *h* ≤ 11, −14 ≤ *k* ≤ 14, −14 ≤ *l* ≤ 14	−12 ≤ *h* ≤ 12, −18 ≤ *k* ≤ 17, −28 ≤ *l* ≤ 30	−11 ≤ *h* ≤ 7, −12 ≤ *k* ≤ 11, −15 ≤ *l* ≤ 14	−11 ≤ *h* ≤ 11, −12 ≤ *k* ≤ 11, −15 ≤ *l* ≤ 15
Reflections collected/unique (*R*_int_)	4240/3434 (0.0413)	14,040/11,155 (0.0271)	4016/3810 (0.0245)	4401/3497 (0.0426)
*D*_c_*/*(Mg·cm^−3^)	1.583	1.609	1.576	1.602
*μ*/mm^−1^	0.807	2.434	9.262	1.671
Data/restraints/parameters	4240/7/335	14,040/168/1093	4016/0/313	4401/0/313
Goodness-of-fit on *F*^2^	1.065	1.115	1.027	1.008
Final *R* [(*I* ≥ 2σ(*I*))] *R*_1_, w*R*_2_	0.0465, 0.1203	0.0407, 0.1176	0.0324, 0.0875	0.0355, 0.0880
R (all data) *R*_1_, w*R*_2_	0.0590, 0.1276	0.0504, 0.1242	0.0341, 0.0885	0.0485, 0.0942
Largest diff. peak & hole/(e·Å^−3^)	0.905 & −0.450	0.513 & −0.516	0.864 & −0.435	0.561 & −0.393
**Compound**	**5**	**6**	**7**	
Chemical formula	C_24_H_24_Co_1.5_N_3_O_11_	C_60_H_58_Co_3_N_6_O_23_	C_52_H_36_Mn_3_N_6_O_12_	
Molecular weight	618.86	1407.91	1101.69	
Crystal system	Monoclinic	Monoclinic	Triclinic	
Space group	*P*2_1_/*n*	*C*2/*c*	*P* − 1	
*a*/Å	6.82955(7)	27.5511(3)	9.6797(6)	
*b/*Å	18.1486(2)	13.9096(2)	10.2210(6)	
*c*/Å	19.02692(18)	15.26792(19)	12.8368(7)	
*α/*(°)	90	90	100.562(5)	
*β*/(°)	94.8812(10)	90.8226(11)	103.381(5)	
*γ/*(°)	90	90	102.206(5)	
*V*/Å^3^	2349.77(4)	5850.44(14)	1171.04(12)	
*Z*	4	4	1	
*F*(000)	1270	2900	561	
Crystal size/mm	0.09 × 0.04 × 0.03	0.05 × 0.03 × 0.02	0.08 × 0.04 × 0.03	
*θ* range for data collection	3.371–67.997	3.203–77.106	2.102–25.496	
Limiting indices	−8 ≤ *h* ≤ 6, −17 ≤ *k* ≤ 21, −22 ≤ *l* ≤ 22	−34 ≤ *h* ≤ 34, −17 ≤ *k* ≤ 17, −11 ≤ *l* ≤ 19	−11 ≤ *h* ≤ 11, −12 ≤ *k* ≤ 12, −15 ≤ *l* ≤ 15	
Reflections collected/unique (*R*_int_)	4273/3958 (0.0357)	5732/4870 (0.0347)	4357/3733 (0.0281)	
*D*_c_*/*(Mg·cm^−3^)	1.749	1.598	1.562	
*μ*/mm^−1^	9.045	7.361	0.870	
Data/restraints/parameters	4273/1/364	5732/0/428	4357/0/331	
Goodness-of-fit on *F*^2^	1.044	1.047	1.022	
Final *R* [(*I* ≥ 2σ(*I*))] *R*_1_, w*R*_2_	0.0321, 0.0790	0.0381, 0.1018	0.0303, 0.0740	
R (all data) *R*_1_, w*R*_2_	0.0354. 0.0804	0.0472, 0.1073	0.0374, 0.0767	
Largest diff. peak & hole/(e·Å^−3^)	0.226 & −0.464	0.333 & −0.433	0.306 & −0.236	

**Table 2:** Knoevenagel condensation of benzaldehyde with malononitrile.

Entry	Catalyst	T(°C)	Time (min)	Catalyst Loading (mol%)	Solvent	Yield *^a^* (%)
1	**4**	25	10	2.0	CH_3_OH	45
2	**4**	25	20	2.0	CH_3_OH	62
3	**4**	25	30	2.0	CH_3_OH	73
4	**4**	25	*40*	*2.0*	CH_3_OH	84
5	**4**	25	50	2.0	CH_3_OH	95
6	**4**	25	60	2.0	CH_3_OH	100
7	**4**	25	60	1.0	CH_3_OH	94
8	**4**	25	60	2.0	H_2_O	98
9	**4**	25	60	2.0	C_2_H_5_OH	95
10	**4**	25	60	2.0	CH_3_CN	88
11	**4**	25	60	2.0	CHCl_3_	65
12	**1**	25	60	2.0	CH_3_OH	89
13	**2**	25	60	2.0	CH_3_OH	100
14	**3**	25	60	2.0	CH_3_OH	100
15	**5**	25	60	2.0	CH_3_OH	86
16	**6**	25	60	2.0	CH_3_OH	82
17	**7**	25	60	2.0	CH_3_OH	100
18	Blank	25	60	−	CH_3_OH	20
19	ZnCl_2_	25	60	2.0	CH_3_OH	32
20	H_3_dpna	25	60	2.0	CH_3_OH	26

*^a^* Calculated by ^1^H NMR spectroscopy: mol(product)/mol(aldehyde + product) × 100.

## Data Availability

Data are contained within the article or supplementary material.

## Supplementary Materials

The following data are available online at https://www.mdpi.com/article/10.3390/molecules28227474/s1. Figure S1: FT-IR spectra; Figure S2: TGA curves; Figure S3: PXRD patterns and additional catalysis data; Figure S4: Accumulation of 2-benzylidenemalononitrile vs. time in the Knoevenagel condensation of benzaldehyde with malononitrile catalyzed by **4**; Figure S5: Catalyst recycling experiments in the Knoevenagel condensation of benzaldehyde with malononitrile catalyzed by **4**; Figure S6: PXRD patterns for **4**: simulated (red), before (black), and after (blue) catalysis; Figure S7: Example of the integration in the ^1^H NMR spectrum of the reaction mixture for the determination of Knoevenagel condensation product (conditions of Table 2, entry 6); Tables S1 and S2: Selected bonding and H-bonding parameters for **1**, **5**, and **6**; Table S3: Substrate scope of catalytic reaction.
