# Supplementary material for: Hydrothermal Assembly, Structural Multiplicity, and Catalytic Knoevenagel Condensation Reaction of a Series of Coordination Polymers Based on a Pyridine-Tricarboxylic Acid
Source: Molecules. 2023 Nov 8;28(22):7474. doi: 10.3390/molecules28227474 (PMC10673224; doi:10.3390/molecules28227474)
Supplement: Supplementary file 1 [file molecules-28-07474-s001.zip › molecules-2676930-supplementary.pdf]

Supplementary Material

Hydrothermal Assembly, Structural Multiplicity, and Catalytic Knoevenagel Condensation Reaction of a Series of Coordination Polymers Based on a Pyridine-tricarboxylic Acid

Xiuqi Kang, Chao Ren, Zhenzhong Mei, Xiaoxiang Fan, Jijun Xue*, Yongliang Shao*, Jinzhong Gu*

State Key Laboratory of Applied Organic Chemistry, College of Chemistry and Chemical Engineering, Lanzhou University, Lanzhou 730000, China; 220220923660@lzu.edu.cn; 416528474@qq.com; meizhzh19@lzu.edu.cn; fanxx21@lzu.edu.cn

* Correspondence: xuejj@lzu.edu.cn (J.X.); shaoylc@lzu.edu.cn (Y.S.); gujzh@lzu.edu.cn (J.G.); Tel.: +86-931-8912589 (J.X.); +86-931-8912500 (Y.S.); +86-931-8915196 (J.G.)

Supplementary material contains:

Figure S1. FT-IR spectra of compounds **1–7**.

Figure S2. TGA curves for **1–7**.

Figure S3. Powder X-ray diffraction for **1–7**.

Figure S4. Accumulation of 2-benzylidenemalononitrile vs. time in the Knoevenagel condensation of benzaldehyde with malononitrile catalysed by **4**. Reaction conditions are those of Table 2, entries 1−6.

Figure S5. Catalyst recycling experiments in the Knoevenagel condensation of benzaldehyde with malononitrile catalysed by **4**. Reaction conditions are those of Table 2, entry 6.

Figure S6. PXRD patterns for **4**: simulated (red), before (black) and after (blue) catalysis.

Figure S7. Example of the integration in the ^{1}H NMR spectrum of the reaction mixture for the determination of Knoevenagel condensation product (conditions of Table 2, Entry 6).

Table S1. Selected bond lengths (Å) and bond angles (°) for **1–7**.

Table S2 Hydrogen bonds in crystal packing [Å, °] for **1**, **5** and **6**..

Table S3 Knoevenagel condensation of substituted benzaldehydes with malononitrile catalyzed by **4**.

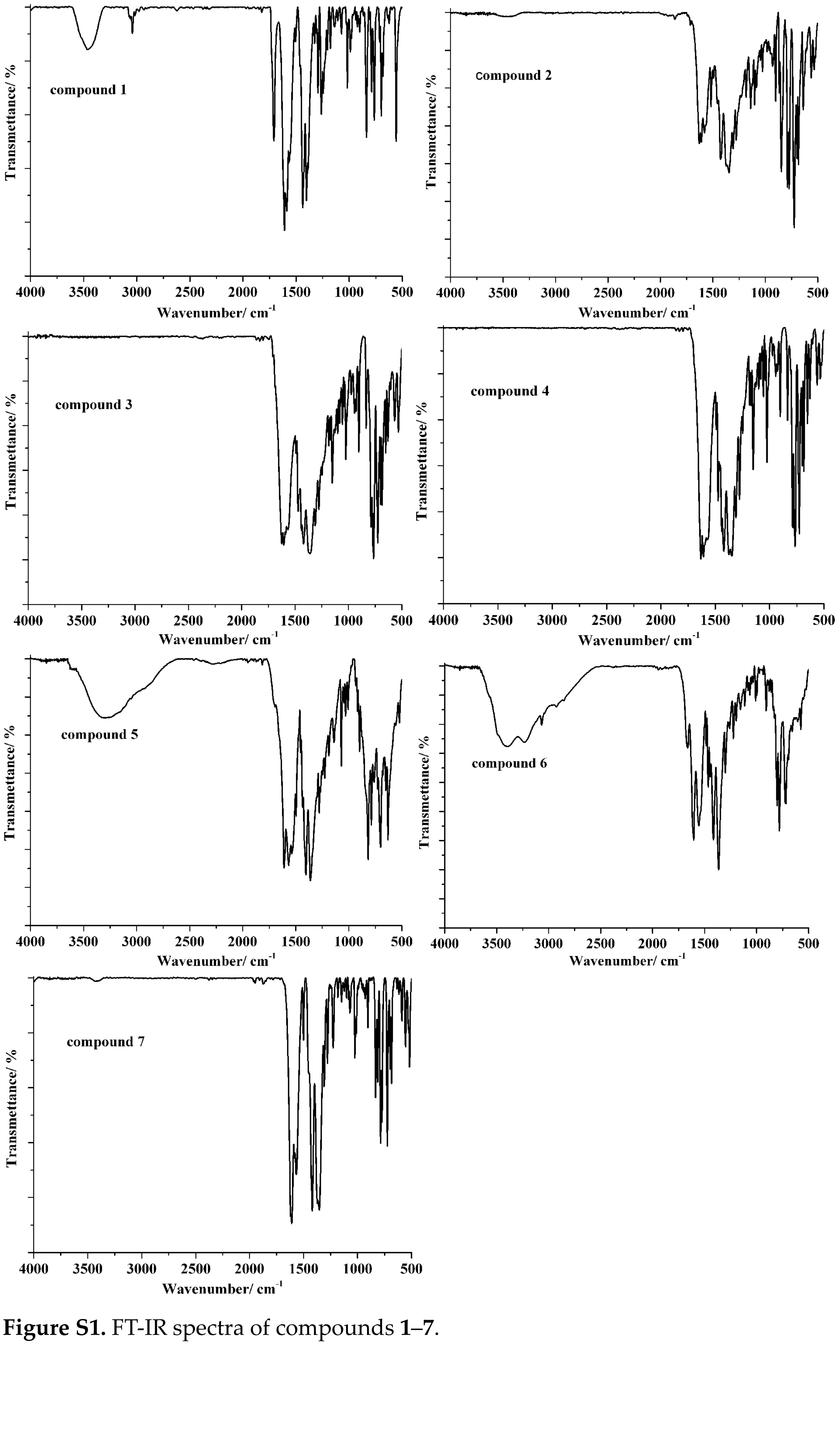

Figure S1. FT-IR spectra of compounds **1**–**7**.

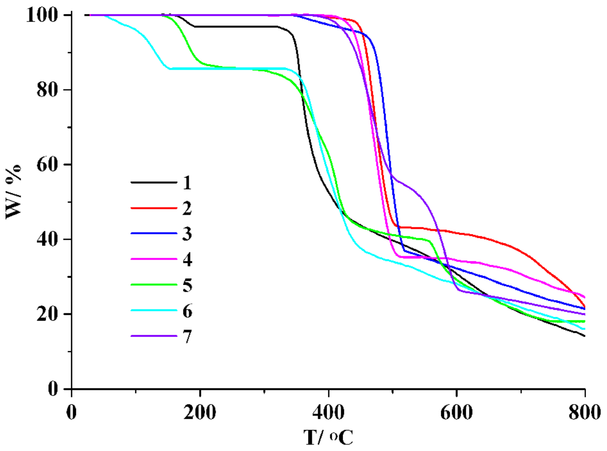

Figure S2. TGA plots of compounds **1–7.**

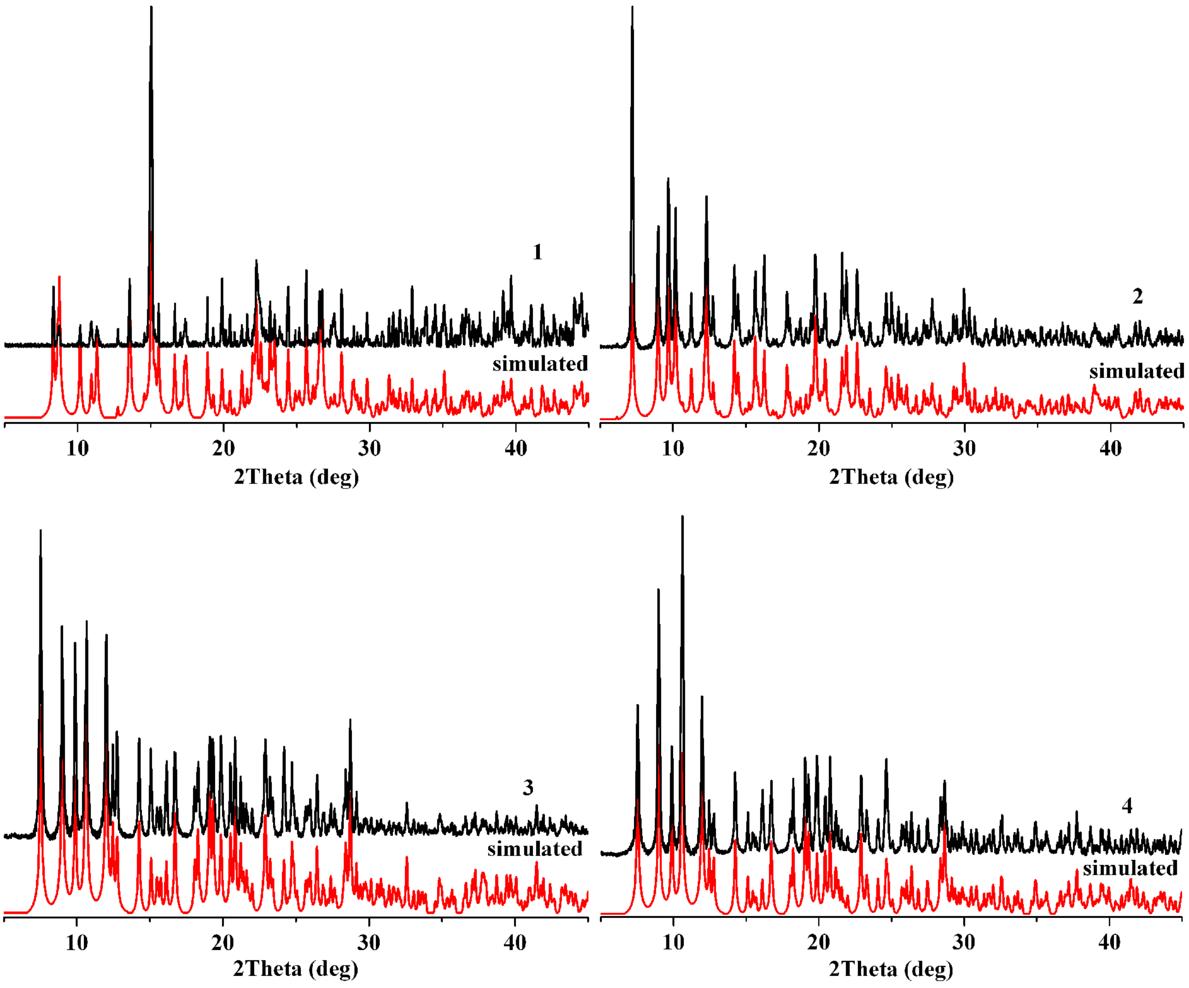

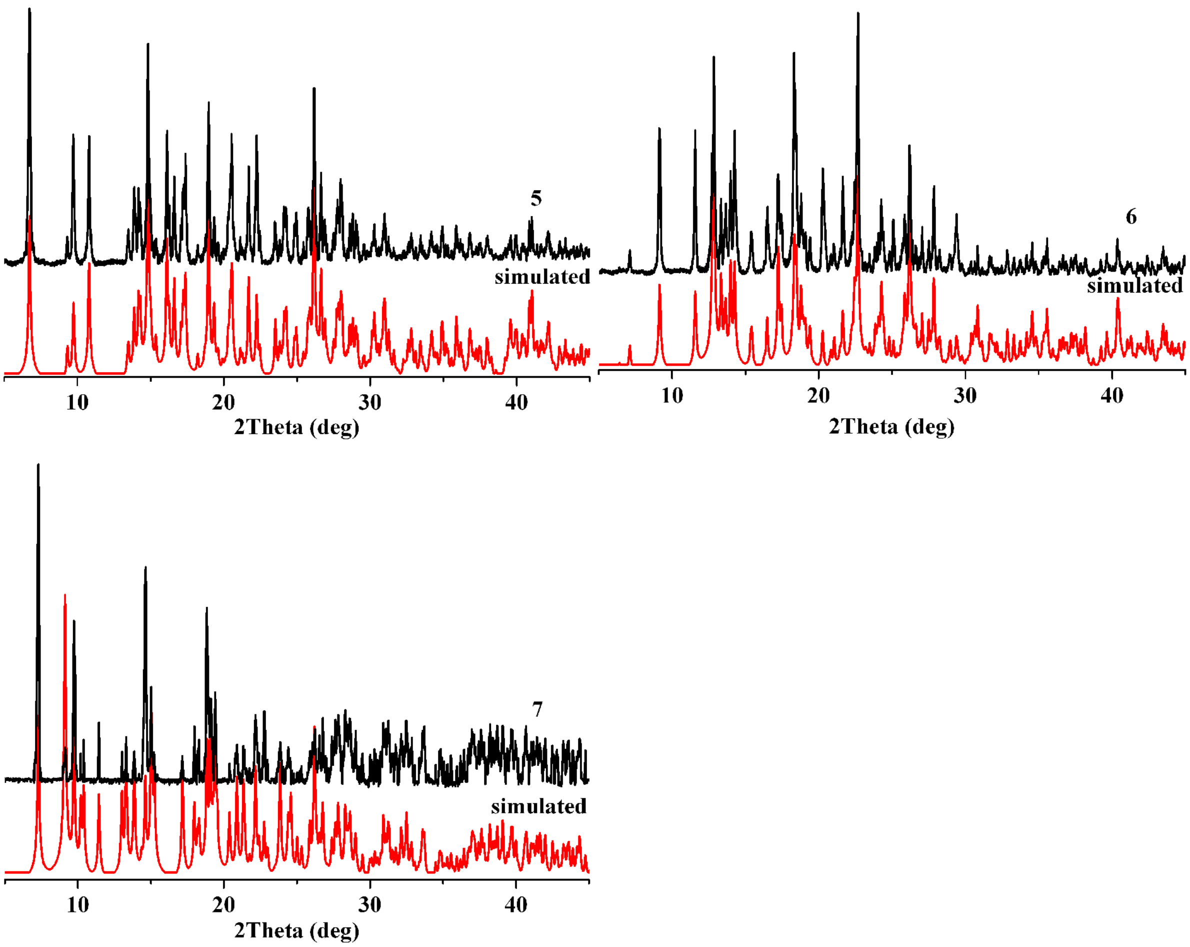

Figure S3. PXRD patterns of compounds **1**–**7** at room temperature.

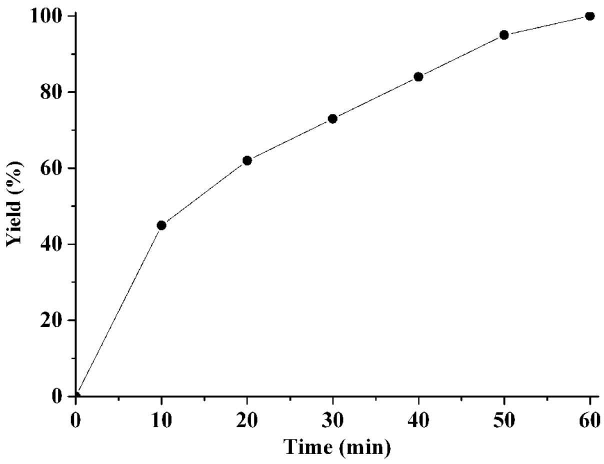

Figure S4. Accumulation of 2-benzylidenemalononitrile vs. time in the Knoevenagel condensation of benzaldehyde with malononitrile catalysed by **4**. Reaction conditions are those of Table 2, entries 1–6.

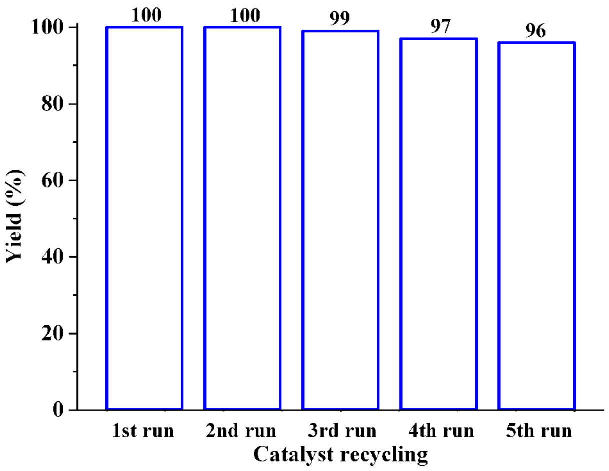

Figure S5. Catalyst recycling experiments in the Knoevenagel condensation of benzaldehyde with malononitrile catalysed by **4**. Reaction conditions are those of Table 2, entry 6.

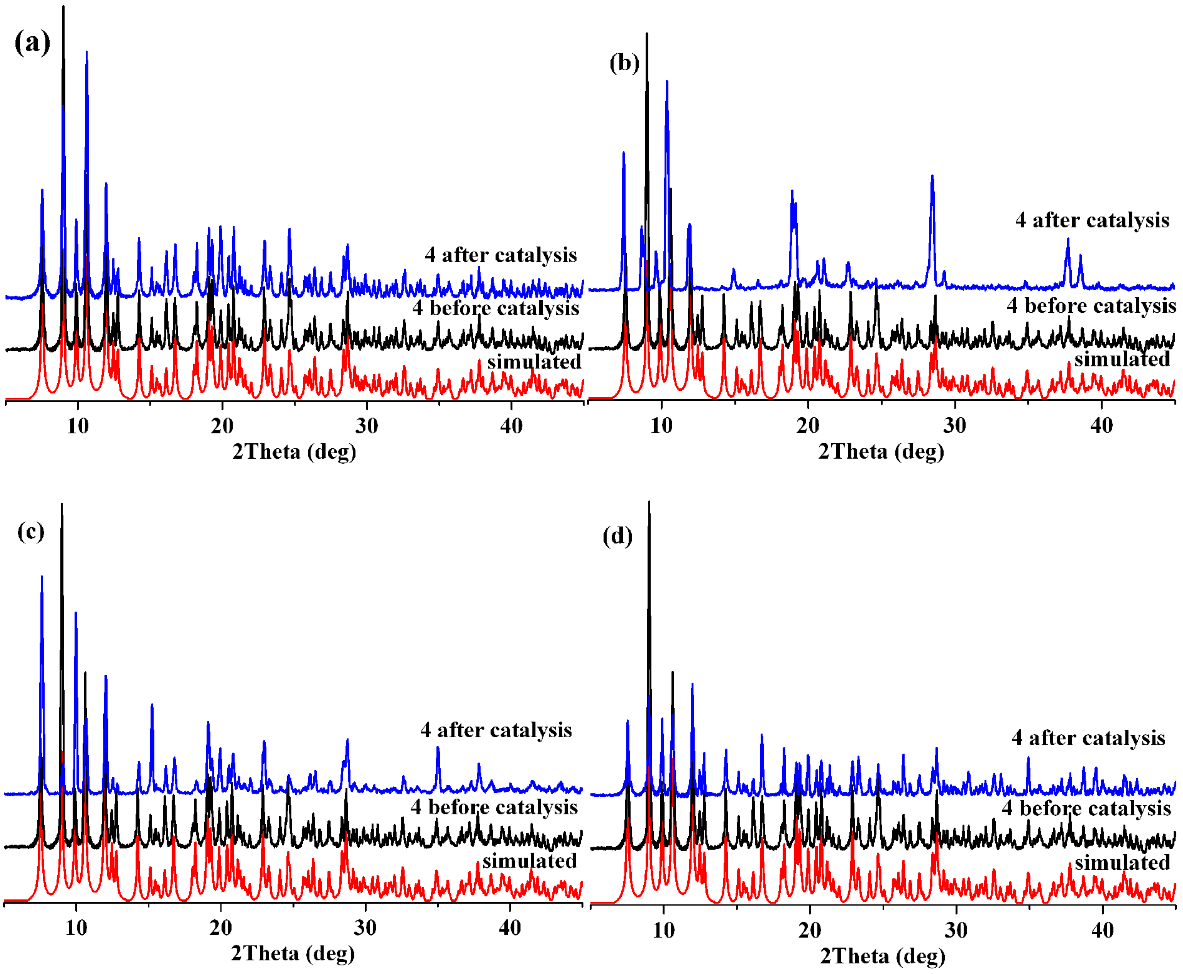

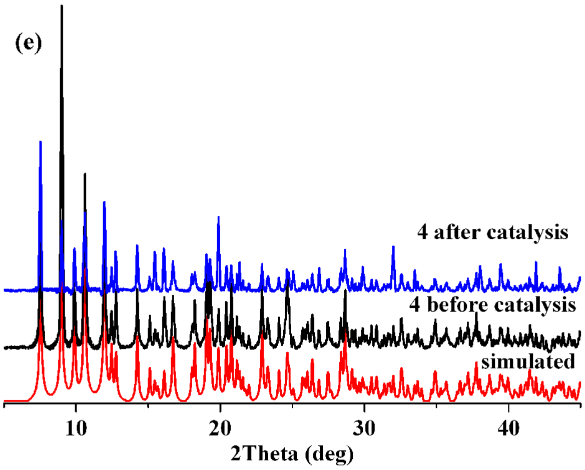

Figure S6. PXRD patterns for **4**: simulated (red), before (black) and after (blue) catalysis. (a) in CH_3OH, (b) in H_2O, (c) in C_2H_5OH, (d) in CH_3CN, (e) in $CHCl_3$.

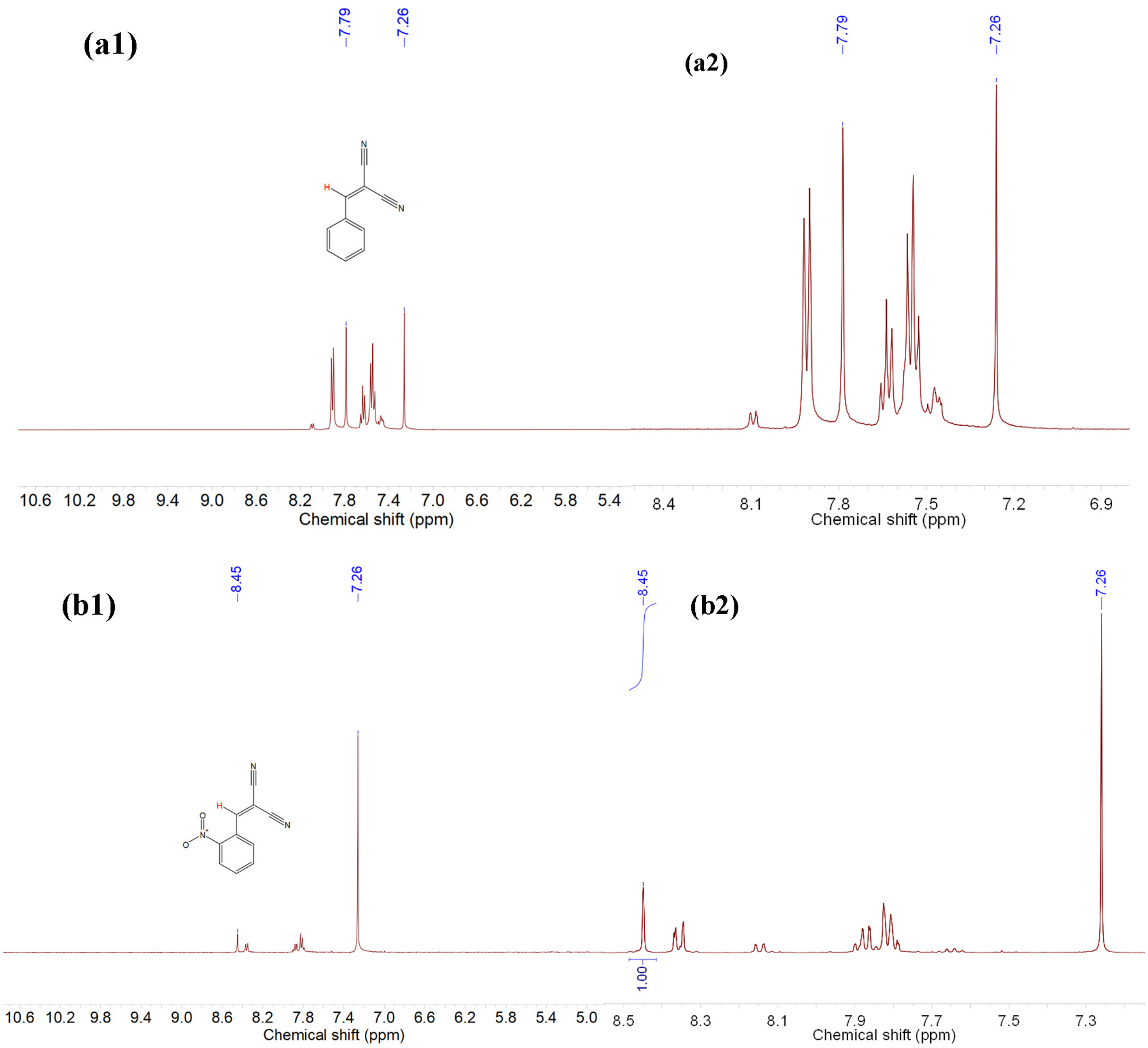

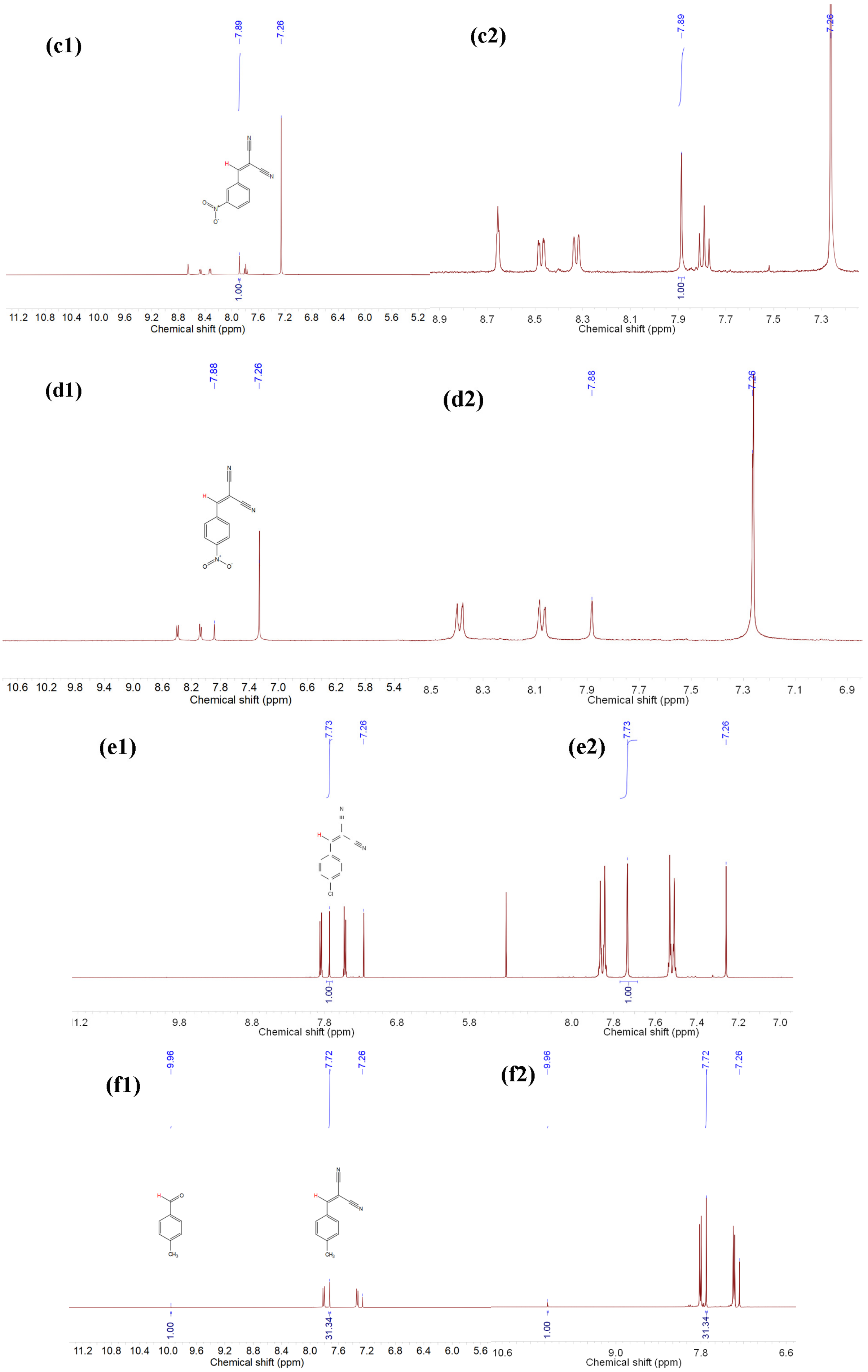
(c1)
7.89
7.26
1.00
11.2 10.8 10.4 10.0 9.6 9.2 8.8 8.4 8.0 7.6 7.2 6.8 6.4 6.0 5.6 5.2
Chemical shift (ppm)
(c2)
7.89
7.26
1.00
8.9 8.7 8.5 8.3 8.1 7.9 7.7 7.5 7.3
Chemical shift (ppm)
(d1)
7.88
7.26
10.6 10.2 9.8 9.4 9.0 8.6 8.2 7.8 7.4 7.0 6.6 6.2 5.8 5.4
Chemical shift (ppm)
(d2)
7.88
7.26
8.5 8.3 8.1 7.9 7.7 7.5 7.3 7.1 6.9
Chemical shift (ppm)
(e1)
7.73
7.26
Cl
1.00
11.2 9.8 8.8 7.8 6.8 5.8
Chemical shift (ppm)
(e2)
7.73
7.26
1.00
8.0 7.8 7.6 7.4 7.2 7.0
Chemical shift (ppm)
(f1)
9.96
7.72
7.26
1.00
31.34
11.2 10.8 10.4 10.0 9.6 9.2 8.8 8.4 8.0 7.6 7.2 6.8 6.4 6.0 5.6
Chemical shift (ppm)
(f2)
9.96
7.72
7.26
1.00
31.34
10.6 9.0 7.8 6.6
Chemical shift (ppm)

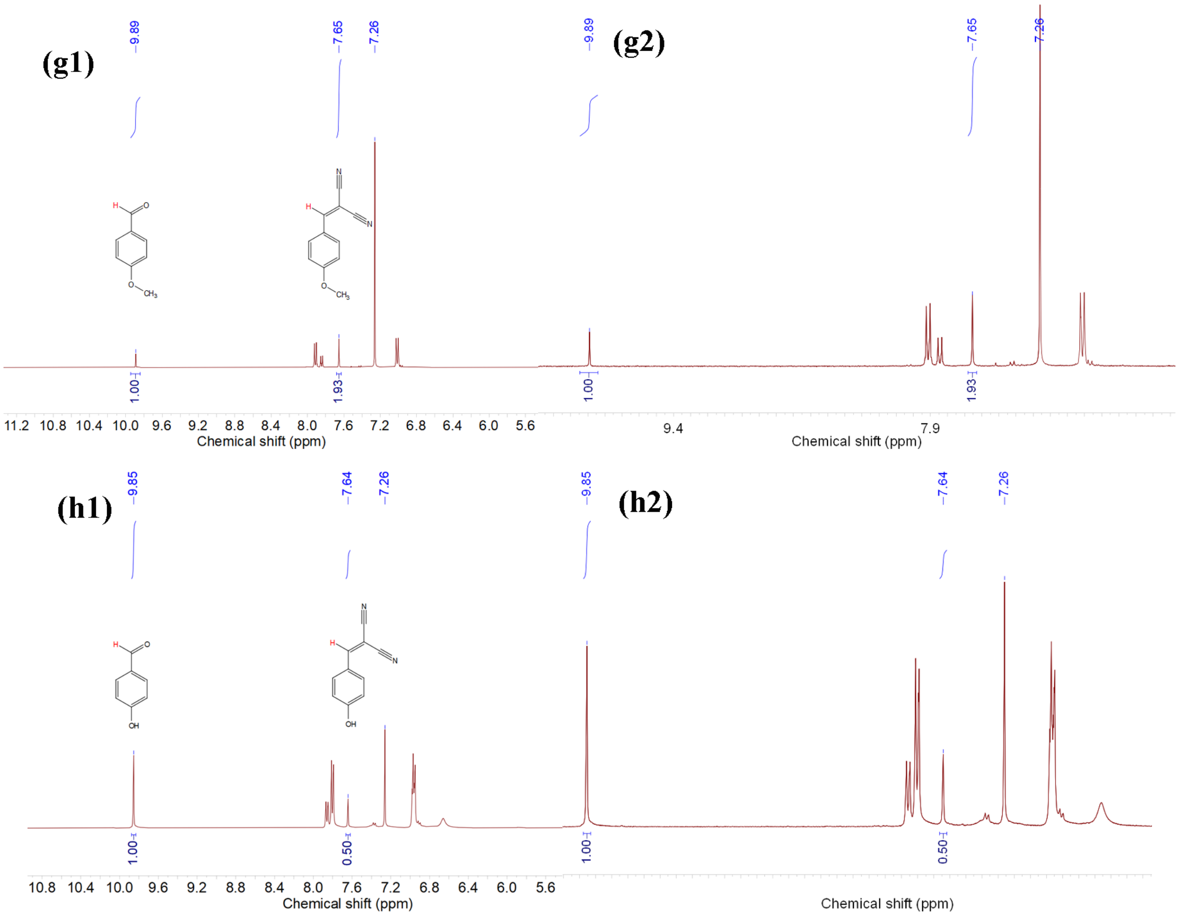

Figure S7. Example of the integration in the ¹H NMR spectrum of the reaction mixture for the determination of Knoevenagel condensation product. (a) Conditions of Table 2, Entry 6).

Product yield calculation in the Knoevenagel condensation reaction. The C(=O)*H* signal of benzaldehyde (substrate) appears at *δ* 10.02 ppm, while 2-benzylidenemalononitrile (product) shows a characteristic signal at *δ* 7.79 ppm.

Total integration of both signals: unreacted benzaldehyde + 2-benzylidenemalononitrile = 0 + 1.00 = 1.00.

Percentage of the unreacted substrate: 0/1.00 = 0%

Conversion of benzaldehyde = yield of 2-benzylidenemalononitrile = 100 − 0 = 100%.

(b) Conditions of Table S3, entry 2.

*Product yield calculation in the Knoevenagel condensation reaction.*The C(=O)*H* signal of 2-nitrobenzaldehyde (substrate) appears at *δ* 10.40 ppm, while(2-nitrobenzylidene)malononitrile (product) shows a characteristic signal at *δ* 8.45 ppm.

Total integration of both signals: unreacted 2-nitrobenzaldehyde + (2-nitrobenzylidene)malononitrile = 0 + 1.00 = 1.00.

Percentage of the unreacted substrate: 0/1.00 = 0%

Conversion of 2-nitrobenzaldehyde = yield of (2-nitrobenzylidene)malononitrile = 100−0 = 100%.

(c) Conditions of Table S3, entry 3.

*Product yield calculation in the Knoevenagel condensation reaction.*The C(=O)*H* signal of 3-nitrobenzaldehyde (substrate) appears at *δ* 10.03 ppm, while (3-nitrobenzylidene)malononitrile (product) shows a characteristic signal at *δ* 7.89 ppm.

Total integration of both signals: unreacted 3-nitrobenzaldehyde + (3-nitrobenzylidene)malononitrile = 0 + 1.00 = 1.00.

Percentage of the unreacted substrate: 0/1.00 = 0%

Conversion of 3-nitrobenzaldehyde = yield of (3-nitrobenzylidene)malononitrile = 100 − 0 = 100%.

(d) Conditions of Table S3, entry 4.

*Product yield calculation in the Knoevenagel condensation reaction.*The C(=O)*H* signal of 4-nitrobenzaldehyde (substrate) appears at *δ* 10.15 ppm, while (4-nitrobenzylidene)malononitrile (product) shows a characteristic signal at *δ* 7.88 ppm.

Total integration of both signals: unreacted 4-nitrobenzaldehyde + (4-nitrobenzylidene)malononitrile = 0 + 1.00 = 1.00.

Percentage of the unreacted substrate: 0/1.00 = 0%

Conversion of 4-nitrobenzaldehyde = yield of (4-nitrobenzylidene)malononitrile = 100−0 = 100%.

(e) Conditions of Table S3, entry 5.

*Product yield calculation in the Knoevenagel condensation reaction.*The C(=O)*H* signal of 4-chlorobenzaldehyde (substrate) appears at *δ* 9.97 ppm, while (4-chlorobenzylidene)malononitrile (product) shows a characteristic signal at *δ* 7.73 ppm.

Total integration of both signals: unreacted 4-chlorobenzaldehyde + (4-chlorobenzylidene)malononitrile = 0+1.00 = 1.00.

Percentage of the unreacted substrate: 0/1.00 = 0%

Conversion of 4-chlorobenzaldehyde = yield of (4-chlorobenzylidene)malononitrile = 100−0 = 100%.

(f) Conditions of Table S3, entry 6.

*Product yield calculation in the Knoevenagel condensation reaction.*The –CH peak of 4-methylbenzaldehyde (substrate) appears at 9.96 ppm while that of (4-methylbenzylidene)malononitrile (product) can be seen at 7.72 ppm.

Total amount: unreacted substrate (4-methylbenzaldehyde) + formed product (4-methylbenzylidene)malononitrile = 1+31.34 = 32.34

Percentage of the unreacted substrate: 1/32.34 = 3.09%

Conversion of 4-methylbenzaldehyde = yield of (4-methylbenzylidene)malononitrile = 100−3.09 = 96.9%.

(g) Conditions of Table S3, entry 7.

*Product yield calculation in the Knoevenagel condensation reaction.*The –CH peak of 4-methoxybenzaldehyde (substrate) appears at 9.89 ppm while that of (4-methoxybenzylidene)malononitrile (product) can be seen at 7.65 ppm.

Total amount: unreacted substrate (4-methoxybenzaldehyde) + formed product (4-methoxybenzylidene)malononitrile = 1 + 1.93 = 2.93

Percentage of the unreacted substrate: 1/2.93 = 34.1%

Conversion of 4-methoxybenzaldehyde = yield of (4-methoxybenzylidene)malononitrile = 100 − 34.1 = 65.9%.

(h) Conditions of Table S3, entry 8.

Product yield calculation in the Knoevenagel condensation reaction. The –CH peak of 4-hydroxybenzaldehyde (substrate) appears at 9.85 ppm while that of (4-hydroxybenzylidene)malononitrile (product) can be seen at 7.64 ppm.

Total amount: unreacted substrate (4-hydroxybenzaldehyde) + formed product (4-hydroxybenzylidene)malononitrile = 1+0.50 = 1.50

Percentage of the unreacted substrate: 1/1.50 = 66.7%

Conversion of 4-hydroxybenzaldehyde = yield of (4-hydroxybenzylidene)malononitrile = 100 − 66.7 = 33.3%.

Table S1 Selected bond lengths (Å) and bond angles (°) for **1–7**.

		**1**			
Co(1)–O(1)i	2.039(2)	Co(1)–O(2)	2.017(2)	Co(1)–O(5)ii	2.246(2)
Co(1)–O(6)ii	2.169(2)	Co(1)–N(2)	2.154(2)	Co(1)–N(3)iii	2.156(2)
O(1)i–Co(1)–O(2)	118.13(10	O(2)–Co(1)–N(2)	91.10(9)	O(1)i–Co(1)–N(2)	94.67(9)
O(2)–Co(1)–N(3)iii	86.57(9)	O(1)i–Co(1)–N(3)iii	89.34(10)	N(2)–Co(1)–N(3)iii	175.96(10)
O(6)ii–Co(1)–O(2)	152.19(10	O(6)ii–Co(1)–O(1)i	87.94(9)	O(6)ii–Co(1)–N(2)	96.51(9)
O(6)ii–Co(1)–N(3)iii	84.10(9)	O(5)ii–Co(1)–O(2)	94.51(9)	O(5)ii–Co(1)–O(1)i	147.22(9)
O(5)ii–Co(1)–N(2)	87.06(9)	O(5)ii–Co(1)–N(3)iii	89.83(10)	O(5)ii–Co(1)–O(6)ii	59.39(9)
		**2**			
Zn(1)–O(1)	2.029(2)	Zn(1)–O(1)i	2.029(2)	Zn(1)–O(7)	2.109(2)
Zn(1)–O(7)i	2.109(2)	Zn(1)–O(11)ii	2.151(2)	Zn(1)–O(11)iii	2.151(2)
Zn(2)–O(4)iv	2.187(2)	Zn(2)–O(6)	2.116(2)	Zn(2)–O(9)	2.019(2)
Zn(2)–O(13)	2.008(2)	Zn(2)–O(16)v	2.167(2)	Zn(2)–O(18)vi	2.126(2)
Zn(3)–O(4)iv	2.081(2)	Zn(3)–O(5)	2.003(2)	Zn(3)–O(14)	2.004(2)
Zn(3)–N(4)	2.168(4)	Zn(3)–N(5)	2.157(5)	Zn(4)–O(10)vi	2.019(2)
Zn(4)–O(16)iv	2.057(2)	Zn(4)–O(17)	2.017(2)	Zn(4)–N(6)	2.108(2)
Zn(4)–N(7)	2.201(2)	Zn(5)–O(2)i	2.023(2)	Zn(5)–O(8)	2.020(2)
Zn(5)–O(11)ii	2.051(2)	Zn(5)–N(8)	2.214(2)	Zn(5)–N(9)	2.097(2)
O(1)i–Zn(1)–O(7)	97.10(7)	O(1)–Zn(1)–O(7)	82.90(7)	O(1)–Zn(1)–O(11)ii	92.84(7)
O(1)–Zn(1)–O(11)iii	87.16(7)	O(7)–Zn(1)–O(11)ii	86.61(6)	O(1)–Zn(7)–O(11)iii	93.39(6)
O(6)–Zn(2)–O(4)iv	88.17(6)	O(6)–Zn(2)–O(16)v	92.41(7)	O(6)–Zn(2)–O(18)vi	178.58(7)
O(9)–Zn(2)–O(4)iv	92.14(7)	O(9)–Zn(2)–O(6)	81.90(7)	O(9)–Zn(2)–O(16)v	87.02(7)
O(9)–Zn(2)–O(18)vi	96.83(7)	O(13)–Zn(2)–O(4)iv	88.25(7)	O(13)–Zn(2)–O(6)	98.60(7)
O(13)–Zn(2)–O(9)	179.37(6)	O(13)–Zn(2)–O(16)v	92.58(7)	O(13)–Zn(2)–O(18)vi	82.66(7)
O(16)v–Zn(2)–O(4)iv	178.90(5)	O(18)vi–Zn(2)–O(4)iv	92.53(6)	O(18)vi–Zn(2)–O(16)	86.87(7)
O(4)iv–Zn(3)–N(4)	165.05(13	O(4)iv–Zn(3)–N(5)	94.79(18)	O(4)iv–Zn(3)–O(5)	97.06(7)
O(14)–Zn(3)–O(5)	108.91(8)	N(4)–Zn(3)–O(5)	85.23(14)	N(5)–Zn(3)–O(5)	50.38(14)
O(4)iv–Zn(3)–O(14)	102.64(6)	O(14)–Zn(3)–N(4)	90.46(13)	O(14)–Zn(3)–N(5)	94.73(14)
N(5)–Zn(3)–N(4)	76.8(2)	O(10)vi–Zn(4)–O(16)i	107.83(6)	O(10)vi–Zn(4)–N(6)	97.01(8)
O(10)vi–Zn(4)–N(7)	85.97(8)	O(16)iv–Zn(4)–N(6)	150.08(8)	O(16)iv–Zn(4)–N(7)	88.19(8)
O(10)vi–Zn(4)–O(17)	104.13(8)	O(16)iv–Zn(4)–O(17)	97.02(7)	O(17)–Zn(4)–N(6)	92.70(8)
O(17)–Zn(4)–N(7)	166.48(8)	N(6)–Zn(4)–N(7)	76.95(9)	O(11)ii–Zn(5)–O(2)i	109.44(6)
N(8)–Zn(5)–O(2)i	87.40(8)	N(9)–Zn(5)–O(2)i	100.37(8)	O(8)–Zn(5)–O(2)i	102.92(8)
O(8)–Zn(5)–O(11)ii	96.52(7)	O(8)–Zn(5)–N(8)	167.37(8)	O(8)–Zn(5)–N(9)	94.00(8)
O(11)ii–Zn(5)–N(8)	86.69(8)	O(11)ii–Zn(5)–N(9)	145.12(8)	N(8)–Zn(5)–N(9)	76.77(9)
		**3**			
Co(1)–O(1)	2.019(2)	Co(1)–O(3)i	2.046(2)	Co(1)–O(6)ii	2.078(2)
Co(1)–N(2)	2.082(2)	Co(1)–N(3)	2.143(2)	Co(2)–O(2)iv	2.106(2)
Co(2)–O(2)v	2.106(2)	Co(2)–O(4)	2.042(2)	Co(2)–O(4)iii	2.042(2)
Co(2)–O(6)vi	2.134(2)	Co(2)–O(6)vii	2.134(2)		

O(1)–Co(1)–O(3)i	100.30(7)	O(1)–Co(1)–O(6)ii	97.10(6)	O(3)i–Co(1)–O(6)ii	108.29(6)
O(1)–Co(1)–N(2)	96.40(8)	O(3)i–Co(1)–N(2)	103.77(7)	O(6)ii–Co(1)–N(2)	142.17(7)
O(1)–Co(1)–N(3)	171.04(8)	O(3)i–Co(1)–N(3)	86.79(8)	O(6)ii–Co(1)–N(3)	85.71(7)
N(2)–Co(1)–N(3)	76.46(8)	O(4)–Co(2)–O(2)iv	83.74(6)	O(4)–Co(2)–O(2)v	96.26(6)
O(4)–Co(2)–O(6)vi	87.49(6)	O(4)–Co(2)–O(6)vii	92.51(6)	O(2)v–Co(2)–O(6)vi	92.64(6)
O(2)v–Co(2)–O(6)vi	87.36(6)				
			**4**		
Zn(1)–O(1)	2.019(2)	Zn(1)–O(3)i	2.066(2)	Zn(1)–O(5)ii	2.018(2)
Zn(1)–N(2)	2.164(3)	Zn(1)–N(3)	2.100(3)	Zn(2)–O(2)	2.043(2)
Zn(2)–O(2)iii	2.043(2)	Zn(2)–O(3)i	2.134(2)	Zn(2)–O(3)vi	2.134(2)
Zn(2)–O(6)ii	2.116(2)	Zn(2)–O(6)v	2.116(2)		
O(5)ii–Zn(1)–O(1)	101.34(9)	O(5)ii–Zn(1)–O(3)i	95.98(9)	O(3)i–Zn(1)–O(1)	110.69(8)
O(5)ii–Zn(1)–N(3)	94.31(10)	O(3)i–Zn(1)–N(3)	139.87(9)	O(1)–Zn(1)–N(3)	105.06(10)
O(5)ii–Zn(1)–N(2)	166.70(10	O(3)i–Zn(1)–N(2)	86.05(10)	O(1)–Zn(1)–N(2)	90.15(10)
O(6)ii–Zn(2)–O(2)	83.20(8)	O(6)v–Zn(2)–O(2)	96.80(8)	O(3)i–Zn(2)–O(2)	87.77(8)
O(3)iv–Zn(2)–O(2)	92.23(8)	O(6)ii–Zn(2)–O(3)i	93.46(8)	O(6)v–Zn(2)–O(3)i	86.54(8)
			**5**		
Co(1)–O(1)	2.082(2)	Co(1)–O(1)i	2.082(2)	Co(1)–O(7)	2.078(2)
Co(1)–O(7)i	2.078(2)	Co(1)–N(3)ii	2.160(2)	Co(1)–N(3)iii	2.160(2)
Co(2)–O(4)	2.088(2)	Co(2)–O(8)	2.131(2)	Co(2)–O(9)	2.119(2)
Co(2)–O(10)	2.126(2)	Co(2)–N(1)iv	2.157(2)	Co(2)–N(2)	2.152(2)
O(1)–Co(1)–O(7)	91.02(6)	O(1)–Co(1)–O(7)i	88.98(6)	N(3)ii–Co(1)–O(7)	89.77(7)
N(3)iii–Co(1)–O(7)	90.23(7)	N(3)ii–Co(1)–O(1)	89.93(6)	N(3)iii–Co(1)–O(1)	90.07(6)
O(4)–Co(2)–O(9)	172.44(7)	O(4)–Co(2)–O(10)	89.30(6)	O(9)–Co(2)–O(10)	97.36(6)
O(4)–Co(2)–O(8)	86.69(6)	O(9)–Co(2)–O(8)	86.56(6)	O(8)–Co(2)–O(10)	175.74(6)
O(4)–Co(2)–N(2)	96.54(7)	O(9)–Co(2)–N(2)	87.16(7)	O(10)–Co(2)–N(2)	89.35(7)
O(8)–Co(2)–N(2)	92.51(7)	O(4)–Co(2)–N(1)iv	92.75(7)	O(9)–Co(2)–N(1)iv	84.16(7)
O(10)–Co(2)–N(1)iv	86.18(7)	O(8)–Co(2)–N(1)iv	92.59(7)	N(2)–Co(2)–N(1)iv	169.64(7)
			**6**		
Co(1)–O(1)	2.061(2)	Co(1)–O(3)i	2.194(2)	Co(1)–O(4)i	2.150(2)
Co(1)–O(7)	2.091(2)	Co(1)–O(8)	2.094(2)	Co(1)–N(1)ii	2.102(2)
Co(2)–O(9)	2.048(3)	Co(2)–O(10)	2.129(2)	Co(2)–O(10)iii	2.129(2)
Co(2)–O(11)	2.103(3)	Co(2)–N(2)	2.141(2)	Co(2)–N(2)iii	2.141(2)
O(3)i–Co(1)–O(4)i	60.65(6)	O(1)–Co(1)–O(4)i	84.65(6)	O(3)i–Co(1)–O(1)	90.56(7)
O(1)–Co(1)–O(7)	91.50(6)	O(1)–Co(1)–O(8)	179.10(6)	O(1)–Co(1)–N(1)ii	91.25(8)
O(7)–Co(1)–O(4)i	162.93(7)	O(7)–Co(1)–O(3)i	102.87(6)	O(7)–Co(1)–O(8)	87.65(7)
O(7)–Co(1)–N(1)ii	98.66(7)	O(4)i–Co(1)–O(8)	96.08(7)	O(3)i–Co(1)–O(8)	89.35(7)
O(8)–Co(1)–N(1)ii	89.16(8)	O(4)i–Co(1)–N(1)ii	98.04(7)	O(3)i–Co(1)–N(1)ii	158.33(6)
O(11)–Co(2)–O(10)	85.34(6)	O(11)–Co(2)–O(10)iii	85.34(6)	O(11)–Co(2)–N(2)	91.61(6)
O(11)–Co(2)–N(2)iii	91.61(6)	O(10)–Co(2)–O(10)iii	170.68(12)	O(10)–Co(2)–N(2)	92.93(7)
O(10)–Co(2)–N(2)iii	87.33(7)	O(9)–Co(2)–O(11)	180.0	O(9)–Co(2)–O(10)	94.66(6)
O(9)–Co(2)–O(10)iii	94.66(6)	O(9)–Co(2)–N(2)	88.39(6)	O(9)–Co(2)–N(2)iii	88.39(6)

N(2)–Co(2)–N(2)iii	176.78(12				
		**7**			
Mn(1)–O(1)	2.118(2)	Mn(1)–O(4)i	2.112(2)	Mn(1)–O(5)ii	2.178(2)
Mn(1)–N(2)	2.207(2)	Mn(1)–N(3)ii	2.313(2)	Mn(2)–O(2)iv	2.170(2)
Mn(2)–O(2)v	2.170(2)	Mn(2)–O(3)	2.139(2)	Mn(2)–O(3)iii	2.139(2)
Mn(2)–O(5)vi	2.120(2)	Mn(2)–O(5)vii	2.120(2)		
O(4)i–Mn(1)–O(1)	105.04(7)	O(4)i–Mn(1)–O(5)ii	111.55(6)	O(1)–Mn(1)–O(5)ii	93.21(6)
O(4)i–Mn(1)–N(2)	94.70(7)	O(1)–Mn(1)–N(2)	85.92(7)	O(5)ii–Mn(1)–N(2)	152.93(6)
O(4)i–Mn(1)–N(3)ii	89.27(7)	O(1)–Mn(1)–N(3)ii	164.84(7)	O(5)ii–Mn(1)–N(3)ii	85.94(6)
N(2)–Mn(1)–N(3)ii	88.00(7)	O(2)iv–Mn(2)–O(3)	97.10(6)	O(2)v–Mn(2)–O(3)	82.90(6)
O(5)vi–Mn(2)–O(3)	95.57(6)	O(5)vii–Mn(2)–O(3)	84.43(6)	O(2)iv–Mn(2)–O(5)vi	91.15(6)
O(2)iv–Mn(2)–O(5)vii	88.85(6)				

Symmetry codes: (**1**) i: –*x*, –*y*+2, –*z*+1; ii: *x*, *y*+1, *z*; iii: *x*–1, *y*, *z*–1; (**2**) i: –*x*+2, –*y*+2, –*z*+2; ii: *x*+1, *y*, *z*; iii: –*x*+1, –*y*+2, –*z*+2; iv: *x*–1, *y*, *z*; v: –*x*+1, –*y*+1, –*z*+1; vi: –*x*, –*y*+1, –*z*+1; (**3**) i: *x*–1, *y*+1, *z*; ii: *x*, *y*+1, *z*; iii: –*x*+2, –*y*, –*z*+1; iv: –*x*+1, –*y*+1, –*z*+1; v: *x*+1, *y*–1, *z*; vi: *x*+1, *y*, *z*; vii: –*x*+1, –*y*, –*z*+1; (**4**) i: *x*+1, *y*, *z*; ii: *x*+1, *y*–1, *z*; iii: –*x*+2, –*y*+1, –*z*+1; iv: –*x*+1, –*y*+1, –*z*+1; v: –*x*+1, –*y*+2, –*z*+1; (**5**) i: –*x*+2, –*y*, –*z*+1; ii: *x*+1, *y*–1, *z*; iii: –*x*+1, –*y*+1, –*z*+1; iv: *x*–1/2, –*y*+1/2, *z*–1/2; (**6**) i: *x*, –*y*+2, *z*–1/2; ii: –*x*+1/2, *y*–1/2, –*z*+1/2; iii: –*x*, *y*, –*z*+1/2; (**7**): i: *x*+1, *y*+1, *z*; ii: *x*, *y*+1, *z*; iii: –*x*, –*y*, –*z*+1; iv: *x*–1, *y*–1, *z*; v: –*x*+1, –*y*+1, –*z*+1; vi: *x*–1, *y*, *z*; vii : –*x*+1, –*y*, –*z*+1.

Table S2 Hydrogen bonds in crystal packing [Å, °] for **1**, **5** and **6**.

Compound	D–H···A	*d*(D–H)	*d*(H···A)	*d*(D···A)	∠DHA	Symmetry code
**1**	O(4)–H(4)···O(7)	0.820	1.951	2.756	166.87	*x*, *y*, *z* – 1
	O(7)–H(1W)···N(1)	0.860	2.193	3.043	179.76	*x* – 1, *y* – 1, *z* – 1
	O(7)–H(2W)···O(6)	0.850	2.015	2.865	179.65	
**5**	O(7)–H(1W)···O(5)	0.850	1.818	2.664	172.64	–*x* + 3/2, *y* – 1/2, –*z* + 3/2
	O(7)–H(2W)···O(2)	0.850	1.900	2.748	176.07	–*x* + 1, –*y*, –*z* + 1
	O(8)–H(3W)···O(3)	0.852	2.046	2.843	155.48	*x* – 1, *y* , *z*
	O(8)–H(4W)···O(11)	0.852	1.959	2.780	161.49	–*x*, –*y* + 1, –*z* + 1
	O(9)–H(5W)···O(6)	0.850	2.073	2.911	168.15	–*x*, –*y* + 1, –*z* + 1
	O(9)–H(6W)···O(2)	0.850	1.807	2.656	176.58	–*x* + 1/2, *y* + 1/2, –*z* + 1/2
	O(10)–H(7W)···O(3)	0.851	1.956	2.696	144.77	
	O(10)–H(8W)···O(6)	0.851	2.075	2.907	165.61	–*x* + 1, –*y* + 1, –*z* + 1
	O(11)–H(9W)···O(6)	0.850	1.925	2.743	161.12	
**6**	O(7)–H(7A)···O(2)	0.851	1.835	2.600	148.75	
	O(7)–H(7B)···O(12)	0.851	2.081	2.880	156.13	*x* – 1/2, *y* – 1/2, *z*
	O(8)–H(8A)···O(5)	0.853	1.845	2.672	163.02	–*x* + 1/2, –*y* + 3/2, –*z*
	O(8)–H(8B)···N(3)	0.852	2.259	3.060	156.51	*x* – 1/2, *y* – 1/2, *z*
	O(9)–H(9A)···O(2)	0.856	1.965	2.715	145.62	
	O(9)–H(9B)···O(2)	0.856	2.227	2.715	116.09	–*x*, *y*, –*z* + 1/2

O(10)–H(10A)···O(3)	0.853	2.176	2.769	126.44	
O(10)–H(10B)···N(3)	0.853	2.215	2.981	149.31	$x - 1/2, y + 5/2, z + 1/2$
O(11)–H(11B)···O(6)	0.863	1.973	2.743	147.92	$-x + 1/2, y + 1/2, -z + 1/2$

Table S3 Knoevenagel condensation of substituted benzaldehydes with malononitrile catalyzed by **4**.[a]

Entry	Substituted benzaldehyde	Product Yield[b], %
1	R = H	100
2	R = 2-NO_2	100
3	R = 3-NO_2	100
4	R = 4-NO_2	100
5	R = 4-Cl	100
6	R = 4-CH_3	97
7	R = 4-OCH_3	66
8	R = 4-OH	33

[a] Reaction conditions: aldehyde (0.5 mmol), propanedinitrile (1.0 mmol), catalyst **4** (2.0 mol.%), CH_3OH (1.0 mL), 25 °C. [b] Calculated by ^1H NMR spectroscopy.
